# Dietary Nucleotides as Potential Modulators of Inflammatory and Metabolic Pathways with Implications for Insulin Resistance

**DOI:** 10.3390/ijms27188133

**Published:** 2026-09-12

**Authors:** Renata Karaś, Urszula E. Binduga, Konrad A. Szychowski

**Affiliations:** 1 Renata Karaś Dietetic Practice, Podole 145A, 39-320 Przecław, Poland; 2 Department of Civilization Diseases and Regenerative Medicine, Medical Faculty, University of Information Technology and Management in Rzeszów, 2 Sucharskiego Street, 35-225 Rzeszów, Poland; ubinduga@wsiz.edu.pl; 3 Department of Biotechnology and Cell Biology, Medical Faculty, University of Information Technology and Management in Rzeszów, 2 Sucharskiego Street, 35-225 Rzeszów, Poland; kszychowski@wsiz.edu.pl

**Keywords:** dietary nucleotides, insulin resistance, oxidative stress

## Abstract

Dietary nucleotides and nucleic-acid-derived compounds are considered bioactive nutrients, but their metabolic relevance remains uncertain. This narrative review, supported by a structured literature search, evaluates intestinal handling and evidence on glucose and lipid metabolism, redox homeostasis, immune function, gut barrier regulation, and microbiota. Direct supplementation studies are distinguished from nucleoside/nucleobase evidence, dietary nucleic acids or purine-rich foods, and endogenous extracellular purinergic signaling. Preclinical studies suggest defined nucleotide preparations may modulate AMP-activated protein kinase (AMPK), insulin receptor substrate 1 (IRS-1)/protein kinase B (AKT)/forkhead box protein O1 (FOXO1) signaling, lipid accumulation, and mitochondrial/redox-related endpoints. In contrast, extracellular adenosine triphosphate (ATP), adenosine diphosphate (ADP), and adenosine 5′-monophosphate (AMP) studies provide mechanistic context but do not show that oral supplementation modifies purinergic signaling in humans. Human intervention evidence is sparse and derived from older adults not selected for insulin resistance, type 2 diabetes, or metabolic-dysfunction-associated steatotic liver disease (MASLD). Reductions in homeostatic model assessment of insulin resistance (HOMA-IR) should be interpreted as changes in a surrogate estimate, not proof of therapeutic efficacy. The supplemental doses evaluated in available human intervention studies appear generally well-tolerated, whereas short-term high nucleotide intake can raise circulating uric acid. Dietary nucleotides therefore remain candidate, not established, metabolic or immune therapies.

## 1. Introduction

Chronic low-grade inflammation, oxidative stress, lipotoxicity, impaired insulin signaling, and disturbances in the gut–liver–adipose tissue axis are interrelated mechanisms contributing to obesity-related metabolic dysfunction and insulin resistance [1,2,3,4,5,6,7]. These processes affect glucose and lipid handling in the liver, adipose tissue, and skeletal muscle and provide a rationale for investigating nutritional components that may influence more than one metabolic or inflammatory axis. Although anti-inflammatory dietary patterns can improve selected inflammatory and metabolic markers [8,9], evidence for individual bioactive nutrients remains heterogeneous and requires careful distinction between mechanistic plausibility and clinically demonstrated benefit.

Dietary nucleotides are potential, albeit still insufficiently studied, dietary factors that may influence inflammatory and metabolic processes [10,11]. They are fundamental components of nucleic acids, and the nutritional literature describes exogenous nucleotides as conditionally essential in settings of rapid growth or physiological stress when endogenous synthesis may not fully meet demand [12]. Experimental and limited clinical studies suggest that nucleotide supplementation can modulate selected metabolic, inflammatory, and immune-related outcomes, although the strength of evidence differs substantially by model and preparation [10,11]. Well-designed clinical trials conducted specifically in populations with diagnosed insulin resistance, metabolic-dysfunction-associated steatotic liver disease (MASLD, formerly termed non-alcoholic fatty liver disease), metabolic syndrome, or type 2 diabetes remain scarce.

This review critically summarizes current evidence on the absorption and metabolism of dietary and exogenous nucleotides and evaluates their potential involvement in inflammation, redox homeostasis, glucose and lipid metabolism, gut barrier regulation, and purinergic signaling. Its specific contribution is to evaluate insulin resistance as a translational hypothesis within this broader metabolic and inflammatory evidence base while explicitly separating direct supplementation evidence from evidence concerning nucleosides and nucleobases, dietary nucleic acids or purine-rich foods, and endogenous extracellular purinergic signaling. Throughout the review, exposures are interpreted in five distinct categories: purified 5′-monophosphates; defined nucleotide mixtures, nucleotide-supplemented formulas or feeds, or yeast-derived preparations; nucleosides and nucleobases; dietary nucleic acids or purine-rich foods; and endogenous extracellular purinergic signaling, the last being contextual only. Particular attention is given to the strength, external validity, formulation, dose, and clinical relevance of the available evidence rather than mechanistic plausibility alone.

## 2. Methodology

### Literature Search and Study Selection

This article is a narrative review supported by a structured and reproducible literature search. The search and screening process was reported with reference to the Preferred Reporting Items for Systematic Reviews and Meta-Analyses (PRISMA) 2020 statement and PRISMA-S where applicable [13,14] without reclassifying the article as a systematic review. PubMed and Scopus were searched without publication year restrictions. The initial search was performed on 1 June 2026. Following peer review, the principal search strategy for dietary and exogenous nucleotide evidence was refined to improve specificity for nutritionally relevant exposures while maintaining sensitivity for eligible studies. The complete refined search was repeated on 30 August 2026 to identify newly published or newly indexed studies. The principal search covered metabolic, inflammatory, intestinal, redox, absorption, and safety outcomes. Endogenous extracellular purinergic signaling was searched separately and used only as mechanistic context. The complete PubMed and Scopus search strategies and further methodological details are provided in Supplementary Data S1. No database-level language or publication type filter was applied. A recent Web of Science bibliometric analysis of exogenous nucleotide research was also examined as an external completeness check [15]. Its record count was not incorporated into the present search flow because the database, search fields, eligibility scope, and bibliometric objective differed from those used in this review.

**Figure 1:**
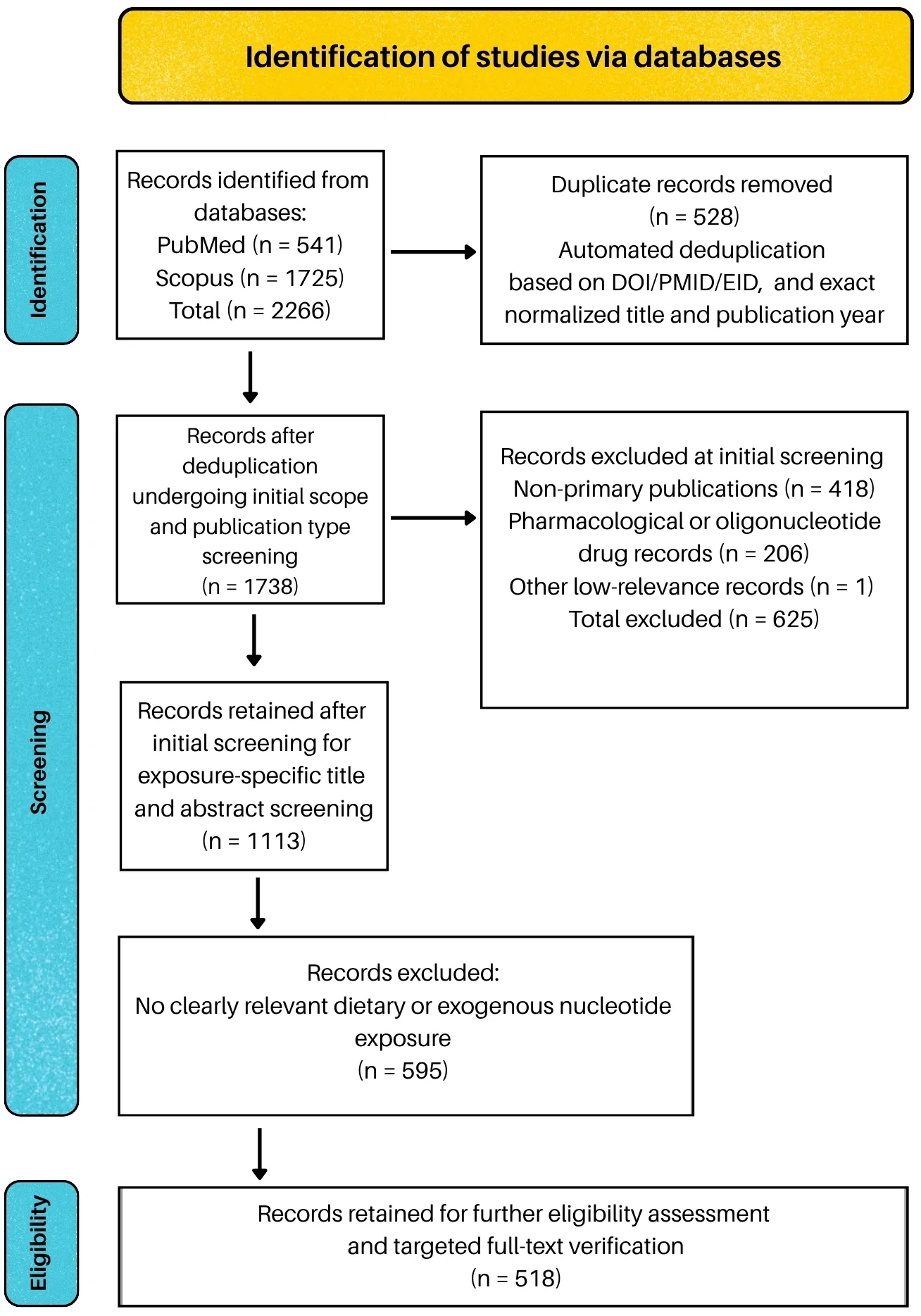
Flow diagram informed by PRISMA 2020 and PRISMA-S for the direct dietary and exogenous nucleotide search based on the updated search performed on 30 August 2026. After staged title and abstract screening, 518 records were retained for further eligibility assessment, with targeted full-text verification performed when required. This number does not represent the number of studies included in the narrative synthesis. Additional sources identified through citation chasing, targeted searches, the separate contextual purinergic signaling search, and methodological or background searches are described in Supplementary Data S1 and were not included in the direct search record counts. Abbreviations include DOI for digital object identifier, EID for Scopus electronic identifier, PMID for PubMed identifier, PRISMA for Preferred Reporting Items for Systematic Reviews and Meta-Analyses, and PRISMA-S for the PRISMA extension for reporting literature searches. The figure was prepared using free online software available at https://www.canva.com/.

Peer-reviewed original human, animal, cellular, analytical, and other relevant experimental or observational studies were eligible when they evaluated nutritionally or experimentally relevant exogenous nucleotide exposure and outcomes within the scope of this review. Non-primary publications, pharmacological nucleotide or nucleoside analogues, oligonucleotide drugs, parenteral interventions without nutritional relevance, and records in which nucleotide terminology referred only to molecular or genetic processes were excluded from the primary evidence set. Nucleosides, nucleobases, and endogenous purinergic signaling studies were retained only as adjacent or contextual mechanistic evidence. The updated direct database search identified 2266 records, including 541 from PubMed and 1725 from Scopus. After 528 duplicate records were removed, 1738 unique records underwent software-assisted title and abstract screening. Initial scope and publication type screening retained 1113 records. A subsequent exposure-specific title and abstract screening step retained 518 records for further eligibility assessment, with targeted full-text verification performed when required. Further eligibility assessment was complemented by backward citation chasing, targeted forward and recent literature checks, and a separate contextual purinergic signaling search, as described in Supplementary Data S1. The final manuscript cites 161 publications. Of these, 152 are original research reports and 9 are secondary, methodological, bibliometric, review, reporting guideline, or consensus and background publications. These bibliography counts are separate from the direct search record flow. Not all 152 original reports originated from the 518 records retained after screening, and they do not represent 152 independent studies because linked publications from the same cohort or experimental program were retained when they contributed additional endpoints. Figure 1 summarizes the direct search and screening flow. Supplementary Data S1 provides the detailed eligibility criteria, deduplication and screening procedures, data extraction variables, citation to claim verification, evidence classification rules, and the origin of sources identified outside of the principal direct search. The 518 records should not be interpreted as the number of studies included in the narrative synthesis.

## 3. Dietary Nucleotides: Sources, Absorption, and Biological Rationale

Due to their biological activity, nucleotides have been incorporated into dietary supplements, fortified foods, and specialized nutritional products. Commercial preparations commonly provide individual 5′-monophosphates, including uridine 5′-monophosphate (UMP) and cytidine 5′-monophosphate (CMP), or defined nucleotide mixtures [16]. Commercial preparations differ substantially in composition and dose, so a single standard adult supplemental intake cannot be inferred from the current literature. Nucleotides have instead been described as conditionally essential under physiological stress, including rapid growth, recovery from injury, infection, and selected disease states, when endogenous synthesis may not fully meet demand [12]. Analytical food composition studies distinguish purine bases, nucleosides, nucleotides, and nucleic acids within foods [17,18,19,20].

Analytical studies have identified nucleotide-, nucleoside-, or purine-related compounds in mushrooms, tea, dairy products, and a broad range of animal- and plant-derived foods, including meat, fish, seafood, legumes, and vegetables [18,19,20,21,22,23]. The measured content varies with food type, origin, processing, and storage [22]. Importantly, many food composition datasets quantify total purines after hydrolysis rather than intact free nucleotides. Such values therefore estimate dietary purine load and should not be interpreted as direct measurements of free nucleotide intake [18,19,20].

Human milk is a well-characterized natural source of free nucleotides and nucleotide-related compounds [24,25,26], and measurable nucleotides are also present in cow’s milk and other dairy products [23]. Human milk composition studies establish exposure but do not by themselves demonstrate functional effects. Evidence for selected infant outcomes instead comes from intervention studies of nucleotide-supplemented formulas, including growth, diarrhea, or immune endpoints and shifts in selected fecal microbial groups [27,28,29,30,31]. This distinction is important because analytical composition, formula fortification, and demonstrated clinical or microbiota outcomes are separate evidence categories. Representative dietary sources and fortified preparations are summarized in Table 1.

**Figure 2:**
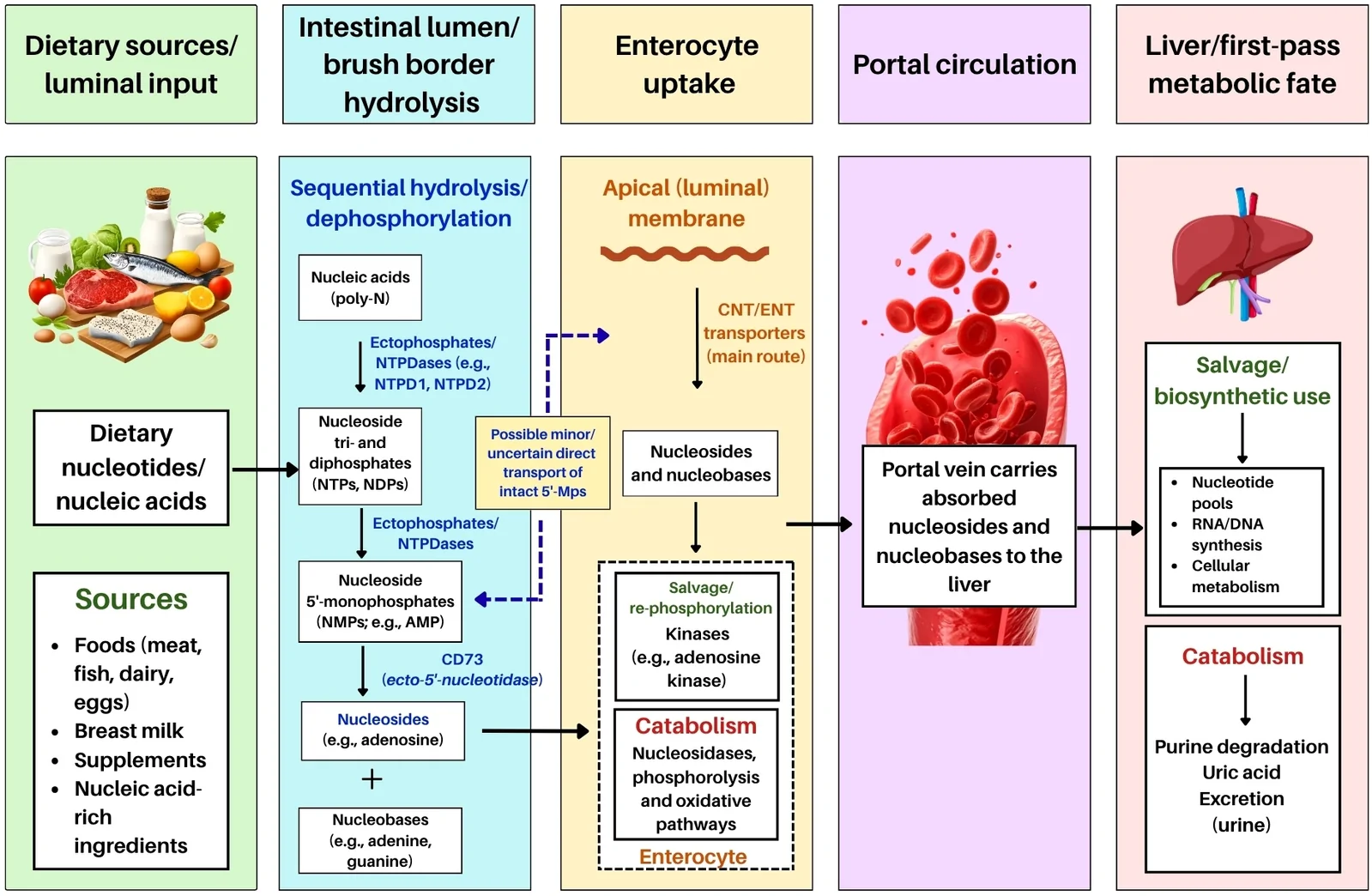
Simplified intestinal processing, absorption, and first-pass metabolic fate of dietary nucleotides and nucleic-acid-derived compounds. Dietary nucleic acids and nucleotides undergo sequential luminal and brush border hydrolysis and dephosphorylation, generating predominantly nucleosides and nucleobases that are taken up by enterocytes, mainly via CNT and ENT transporters. Within enterocytes, absorbed compounds may undergo salvage/re-phosphorylation or catabolism before transfer through the portal circulation to the liver, where they contribute to nucleotide pools and biosynthetic pathways or undergo further degradation, including purine catabolism to uric acid. Solid arrows indicate predominant or experimentally supported metabolic and transport routes, whereas dashed arrows indicate possible, minor, or uncertain pathways, including direct transport of intact 5′-monophosphates. Abbreviations: 5′-MPs, 5′-monophosphates; AMP, adenosine 5′-monophosphate; CD73, ecto-5′-nucleotidase; CNT, concentrative nucleoside transporter; DNA, deoxyribonucleic acid; ENT, equilibrative nucleoside transporter; NDPs, nucleoside diphosphates; NMPs, nucleoside monophosphates; NTPs, nucleoside triphosphates; NTPDase, ectonucleoside triphosphate diphosphohydrolase; poly-N, polynucleotides; RNA, ribonucleic acid. The figure was prepared using free online software available at https://www.canva.com/.

Nucleotides have been proposed as conditionally essential nutrients under selected conditions of rapid growth, tissue repair, intestinal stress, or immune activation when endogenous de novo synthesis may be insufficient or energetically costly [12]. Experimental and clinical data indicate that nucleotide-containing interventions can modulate selected redox and inflammatory outcomes, whereas intestinal barrier and mucosal effects are supported mainly by animal and early-life models [11,35,36,37]. In infant nutrition, different trials support different endpoints. Reported outcomes include reduced diarrhea and selected immune responses [28], growth measures [27], immune outcomes [29,30], and shifts in selected fecal microbial groups [31]. These findings should not be collapsed into a single replicated effect. In adults, nucleotides are often used as components of complex nutritional formulations or combined interventions, whereas isolated effects remain much less well-documented [38]. Piglet intestinal explants provide direct experimental evidence that exogenous nucleic acids and nucleotides are extensively hydrolyzed and taken up predominantly as nucleosides [39]. Consistent mechanistic studies support prior dephosphorylation and subsequent nucleoside transport rather than efficient direct absorption of intact mononucleotides [40,41,42]. Figure 2 summarizes the main steps in intestinal hydrolysis, uptake, and first-pass metabolic handling.

Particular importance has been attributed to the final step of nucleotide hydrolysis, namely the conversion of adenosine 5′-monophosphate (AMP) to adenosine, which is catalyzed by ecto-5′-nucleotidase (CD73) [40]. Kao et al. showed that CD73 is predominantly present on the apical surface of intestinal epithelial cells, suggesting that terminal dephosphorylation of luminal nucleotides may occur close to the brush border of enterocytes [40]. Further support for the importance of prior dephosphorylation comes from the study by Narumi et al., who showed that 2′-deoxyadenosine 5′-monophosphate (dAMP) can serve as a substrate for the concentrative nucleoside transporter 3 (CNT3), but its transport is much less efficient than that of deoxyadenosine, whereas CD73 expression facilitates its uptake by converting dAMP to the corresponding nucleoside before transport [41]. Together, these findings suggest that efficient intestinal uptake of nucleotide-derived compounds usually requires prior dephosphorylation and subsequent transport of nucleosides by concentrative nucleoside transporter (CNT) and equilibrative nucleoside transporter (ENT) families [41]. However, human data on intestinal transport of nucleosides and nucleobases remain limited, and observations from animal models or engineered expression systems cannot always be directly extrapolated to human physiology. Isotope tracing studies provide complementary information on the fate of dietary nucleic-acid-derived material. Berthold et al. fed uniformly 13C-labelled Spirulina-derived nucleic acids to a hen for 4 weeks and four mice for 6 days and then analyzed isotopic enrichment of nucleosides isolated from hepatic ribonucleic acid (RNA) [43]. Pyrimidine nucleosides showed substantially greater intact ribose-plus-base labelling than purines, supporting preferential salvage/incorporation of dietary pyrimidine nucleosides into hepatic RNA. The experiment is important for nutritional salvage biology but should not be cited as evidence that intact dietary 5′-monophosphate nucleotides cross the intestinal epithelium unchanged. The experimental basis for digestion and absorption of nucleotide-derived compounds is summarized in Table 2.

A recent mouse study by Hayashi et al. extended this evidence to hydrolyzed dietary DNA and defined the chemical forms appearing after intestinal passage. Following oral administration of hydrolyzed salmon milt DNA, deoxyribonucleotides, or deoxyribonucleosides, and pyrimidine deoxyribonucleosides were the predominant systemic forms, whereas purine-derived material was extensively catabolized toward uric acid; dinucleotides were also detected in portal blood and on the basolateral side of Ussing chamber preparations [44]. This finding expands the range of detectable absorption products but does not demonstrate efficient uptake of intact dietary 5′-monophosphates.

**Table 2:** Selected experimental evidence on intestinal digestion and absorption of nucleotide-derived compounds.

Process Stage	Experimental Model	Key Enzymes, Transporters, or Mechanistic Observations	Substrate/Product Examined	References
Extracellular hydrolysis at the enterocyte surface	Caco-2 cell line	NTPDase1, NTPDase2, neutral ecto-phosphatase, ecto-5′-nucleotidase	ATP and intermediate products of its hydrolysis	[45]
Final dephosphorylation of AMP to adenosine at the brush border	Intestinal epithelium; cellular model and mice	CD73/ecto-5′-nucleotidase	Dephosphorylation of AMP to adenosine	[40]
Absorption of mononucleotides after prior dephosphorylation	Caco-2 and COS-7 cells	CD73, CNT3	dAMP, dAdo	[41]
Transfer of DNA-derived components into portal circulation	Rat intestinal loop model; Caco-2 cells; human hiPSC-SIEC	Mechanisms of nucleoside transport, with no evidence of efficient direct transport of all dNMPs	DNA-derived components from salmon milt extract	[42]
Transport across the plasma membrane of enterocytes and other cells	Proteoliposome system with recombinant human protein	hENT1	Adenosine	[46]
Dietary nucleic-acid-derived salvage and tissue incorporation	Hen (4 weeks) and mice (6 days) fed uniformly 13C-labelled Spirulina-derived material	Isotope tracing of nucleosides isolated from hepatic RNA; preferential intact pyrimidine nucleoside labelling relative to purines	Dietary nucleic acids → hepatic RNA nucleosides; not evidence of intact 5′-monophosphate absorption	[43]
Absorption of dietary DNA-derived oligomers and monomers	Mouse in vivo; ex vivo Ussing chamber intestinal preparations	Predominant systemic appearance as pyrimidine deoxyribonucleosides; purine catabolism toward uric acid; dinucleotides detectable in portal blood and on the basolateral side	Hydrolyzed salmon milt DNA, deoxyribonucleotides, deoxyribonucleosides, and dinucleotides	[44]

Abbreviations: AMP, adenosine 5′-monophosphate; ATP, adenosine triphosphate; CD73, ecto-5′-nucleotidase; CNT3, concentrative nucleoside transporter 3; COS-7, African green monkey kidney-derived cell line; dAdo, deoxyadenosine; dAMP, 2′-deoxyadenosine 5′-monophosphate; dNMPs, deoxyribonucleoside monophosphates; DNA, deoxyribonucleic acid; hENT1, human equilibrative nucleoside transporter 1; hiPSC-SIEC, human-induced pluripotent stem-cell-derived small intestinal epithelial cells; NTPDase1, ectonucleoside triphosphate diphosphohydrolase 1; NTPDase2, ectonucleoside triphosphate diphosphohydrolase 2; RNA, ribonucleic acid.

## 4. Mechanistic Links Between Dietary Nucleotides, Inflammation, and Metabolic Regulation

### 4.1. Effects on Energy Metabolism

Preclinical studies suggest that dietary or exogenous nucleotides may influence mitochondrial and cellular energy metabolism through effects on nucleotide availability, adenosine triphosphate (ATP) production, AMP-activated protein kinase (AMPK)/sirtuin 1 (SIRT1)/peroxisome proliferator-activated receptor gamma coactivator 1-alpha (PGC-1α) signaling, and redox homeostasis [47,48]. These supplementation-related effects should be distinguished from endogenous extracellular purinergic signaling. After intestinal hydrolysis and absorption, nucleotide-derived nucleosides and nucleobases may support intracellular salvage pathways and nucleotide pools. Extracellular ATP, adenosine diphosphate (ADP), AMP, and their metabolites instead act as local signals whose physiological effects do not establish the consequences of oral nucleotide supplementation.

Evidence for exogenous nucleotides and energy metabolism is predominantly preclinical. In aged SAMP8 mice, long-term supplementation was associated with higher hepatic glycolytic activity and ATP production, lower lipid and fibrosis-related changes, and reduced de novo purine biosynthesis [49]. These results indicate changes in energy-related endpoints, but the aging model limits clinical inference. In palmitate-treated HepG2 cells, exogenous nucleotides increased glucose consumption and glycogen content and modulated PEPCK, G6Pase, IRS-1/AKT/FOXO1, and AMPK signaling [10]. This is relevant to hepatic insulin resistance but cannot be extrapolated to whole-body glucose homeostasis. Cardiac SAMP8 studies likewise reported higher ATP-related bioenergetic markers, citrate synthase and succinate dehydrogenase activity, and AMPK/TFAM-related mitochondrial regulation [47]. Together, these data indicate modulation of mitochondrial bioenergetics in aging tissues. The redox findings from these models are discussed separately in Section 4.3. Related zebrafish evidence links dietary nucleotide or AMP supplementation to reduced hepatic lipid deposition through AMPK activation [50].

A mechanistically distinctive dietary study by Guo et al. fed larval zebrafish a control diet or 0.1% mixed nucleotides for 2 weeks and measured growth, oxygen-consumption-derived standard metabolic rate, and intestinal microbiota [51]. Microbiota pooled from nucleotide-fed fish were transferred to germ-free recipients at 10^6^ CFU/mL. Recipients colonized with the nucleotide-associated microbiota showed an approximately 25% lower standard metabolic rate, whereas short direct exposure of germ-free fish to 0.1% nucleotides for 3 days did not reproduce the energy expenditure effect [51]. This experiment supports a microbiota-mediated component of growth/energy regulation, but it is an aquaculture model and does not establish a comparable mechanism in mammalian insulin resistance.

Direct dietary evidence predating the current SAMP8 literature shows that nucleotide availability can alter hepatic growth, glycogen, protein synthesis, and lipid composition but also demonstrates marked model dependence. Novak et al. fed weanling BALB/c mice for 5 weeks with standard chow, a nucleotide-free semi-purified diet, the same diet containing a five nucleotide mixture at 0.21% (*w*/*w*), or AMP alone at 0.0425% (*w*/*w*) [52]. Relative liver weight and hepatic glycogen were lower, whereas hepatic cholesterol and lipid phosphorus were higher, in nucleotide-deprived mice than in supplemented groups. AMP alone produced a stronger contrast with the nucleotide-free condition than the mixture. In weanling male Wistar rats fed for 4 weeks, three graded levels of AMP, guanosine 5′-monophosphate (GMP), inosine 5′-monophosphate (IMP), UMP, and CMP altered hepatic microsomal long-chain polyunsaturated fatty acids and desaturase activities [53]. In contrast, male Sprague–Dawley rats fed a human-milk-like nucleotide mixture at 1.0% of the diet for 5 weeks showed cortical phospholipid and fatty acid changes but no corresponding difference in hepatic phosphatidylcholine or the phosphatidylcholine-to-phosphatidylethanolamine ratio [54]. A complementary 10-day study in male Wistar rats fed a purified diet with or without nucleotides found lower fractional protein synthesis rates in the liver and small intestine during nucleotide deprivation, together with reduced hepatic RNA/ribosome indices and lower small intestinal DNA [55]. These older studies therefore support biological activity but not a uniform direction of hepatic lipid or protein synthesis effects.

Complementary deprivation and intestinal recovery experiments provide older mechanistic evidence that dietary nucleotide availability can influence intracellular nucleotide pools and mitochondrial recovery. López-Navarro et al. found transient reductions in hepatic ATP, ADP, GTP, CDP, and RNA during nucleotide deprivation, with values returning toward those of nucleotide-supplemented rats by week 3 [56]. In weanling rats recovering from lactose-induced chronic diarrhea, Arnaud et al. reported earlier normalization of ileal mitochondrial ATPase, citrate synthase, and malate dehydrogenase activities with dietary nucleotide supplementation [57]. Both studies support condition-dependent nutritional effects, but neither establishes metabolic benefit above nutritional sufficiency.

In brown adipose tissue, nucleotide supplementation in SAMP8 mice was associated with changes in oxidative stress and thermogenesis-related parameters linked to SIRT1 signaling [58]. In oxidatively stressed rat pheochromocytoma PC-12 cells, an exogenous nucleotide mixture reduced ROS and MDA, increased ATP and mitochondrial membrane potential, and activated the oxidized nicotinamide adenine dinucleotide (NAD^+^)/SIRT1/PGC-1α pathway [59]. Dietary AMP also increased whole-body energy expenditure and AMPK-related metabolic activity in mice [60], although findings obtained with a single monophosphate should be distinguished from those obtained with mixed nucleotide preparations. Studies that manipulate nucleotide biosynthesis or adenylate kinase 2 further support the importance of nucleotide homeostasis for mitochondrial metabolism, but they do not directly test dietary supplementation [61,62].

More recent work extended this aging model evidence to skeletal muscle. Wu et al. studied SAMP8 mice beginning at 3 months of age with prolonged nucleotide supplementation assessed after 9 or 15 months and complementary hydrogen peroxide (H_2_O_2_)-stressed mouse C2C12 myotube experiments [63]. Supplementation attenuated age-related losses in muscle mass and strength and was associated with changes in protein turnover and metabolomic pathways. This broadens the tissue-level evidence but remains an aging/sarcopenia program from the same wider nucleotide supplementation research network and does not establish improved insulin action in humans.

### 4.2. Extracellular Purinergic Signaling as Contextual Mechanistic Evidence

The following studies concern endogenous extracellular nucleotide release, receptor activation, and ectonucleotidase activity. They provide mechanistic context for metabolic regulation but should not be interpreted as direct evidence of the effects of dietary nucleotide supplementation. At the intestinal epithelium, extracellular ATP is sequentially hydrolyzed by ectonucleotidases, thereby shaping local ATP, ADP, AMP, and adenosine availability [45].

In the liver, Tatsushima et al. showed that high glucose promotes vesicular nucleotide transporter (VNUT)-dependent ATP release from hepatocytes, which contributes to triglyceride secretion and inflammatory responses [64]. Inhibition or loss of VNUT activity was associated with reduced triglyceride levels and protection against pathological changes related to non-alcoholic steatohepatitis in experimental models [64]. Additional support for the relevance of extracellular purine signaling in steatotic liver disease comes from the study by Tokumaru et al., who used hepatocyte-specific, genetically encoded G-protein-coupled receptor-activation-based (GRAB) sensors to visualize extracellular ATP and adenosine dynamics in zebrafish models of alcoholic and metabolic steatotic liver disease [65]. The authors observed disease-specific changes in hepatic extracellular ATP and adenosine levels during progression, and clodronate treatment, which reduced extracellular ATP and adenosine, alleviated the MASLD-like phenotype. Together, these observations indicate that extracellular purine metabolites may participate in steatotic liver disease progression, including inflammatory and fibrotic processes. However, this evidence concerns extracellular purinergic signaling and should not be interpreted as direct evidence of dietary nucleotide supplementation [65]. More recently, Ma et al. showed that ectonucleoside triphosphate diphosphohydrolase 5 (ENTPD5)-mediated hydrolysis of extracellular ATP to ADP in hepatocytes is important for maintaining hepatic glucose and lipid metabolism and for supporting brown adipose tissue thermogenesis [66]. Inhibition of ENTPD5 increased adrenomedullin expression and secretion, reduced uncoupling protein 1 (UCP1) expression in brown adipose tissue, and promoted obesity, MASLD, and diabetes-like metabolic disturbances in experimental models [66]. The ENTPD5 results also show that the metabolic relevance of extracellular nucleotides is not limited to receptor activation but depends on ectonucleotidase-mediated control of ATP and ADP concentrations in the extracellular microenvironment.

Purinergic signaling also contributes to tissue-specific regulation of glucose metabolism in the intestine and skeletal muscle. Arguin et al. showed that loss of P2X7 receptor expression increased intestinal glucose transit and was associated with hepatic steatosis [67]. Mechanistically, activation of P2X7 promotes internalization of glucose transporter type 2 (GLUT2), thereby reducing the capacity of enterocytes to transport glucose [67]. In contrast, Osorio-Fuentealba et al. demonstrated that ATP released from skeletal muscle cells in response to electrical stimulation increased glucose transporter type 4 (GLUT4) translocation and glucose uptake through the phosphoinositide 3-kinase gamma (PI3Kγ)–AKT–AKT substrate of 160 kDa (AS160) pathway, including under insulin-resistant conditions [68]. The effects of extracellular ATP are tissue-specific. It may limit intestinal glucose transport while promoting glucose uptake in skeletal muscle [67,68].

The relevance of extracellular nucleotide metabolism has also been demonstrated at the whole-organism level [69,70]. Sandhu et al. found that global deletion of ectonucleoside triphosphate diphosphohydrolase 3 (NTPDase3), an enzyme involved in extracellular nucleotide hydrolysis, protected mice against diet-induced obesity and obesity-associated glucose intolerance by increasing basal energy metabolism [69]. This indicates that altering the rate of extracellular ATP/ADP degradation can influence the whole-body metabolic phenotype. Zhang et al. showed that the P2Y2 receptor, which is activated by ATP and uridine triphosphate (UTP), promotes high-fat-diet-induced obesity by affecting adipogenesis, adipose tissue inflammation, and adipocyte metabolism [70]. Qian et al. further demonstrated that the P2Y2 receptor participates in terminal adipocyte differentiation and may promote insulin resistance by suppressing AKT signaling [71]. It is also important to note that extracellular nucleotide signals can be further converted into purine metabolites with independent metabolic activity. Niemann et al. showed that apoptotic brown adipocytes release purine metabolites, particularly inosine [72]. Inosine increased thermogenic gene expression and oxygen consumption in brown adipocytes through purinergic P1 receptor-dependent activation of cyclic AMP/protein kinase A (PKA) signaling. The authors also showed that equilibrative nucleoside transporter 1 (ENT1) controls extracellular inosine levels. ENT1 blockade or deletion increased brown adipose tissue activity and protected against diet-induced obesity in experimental models [72]. Although inosine is a nucleoside rather than a nucleotide, these findings suggest that the metabolic relevance of extracellular purine signaling extends beyond ATP and includes downstream purine metabolites [72]. Additional evidence comes from studies in weaned piglets, in which exogenous nucleotide supplementation influenced hepatic enzymes involved in energy metabolism, including pyruvate kinase and pyruvate carboxylase, as well as the expression of genes related to energy and glycolipid metabolism, including citrate synthase [73]. These data support the concept that exogenous nucleotides may contribute to metabolic regulation in rapidly growing animals, although their relevance to adult human metabolic disease remains uncertain. Table 3 summarizes selected contextual mechanisms of extracellular purinergic signaling.

### 4.3. Redox Homeostasis and Oxidative Stress Outcomes

#### 4.3.1. Cellular and Animal Evidence

Oxidative stress reflects an imbalance between reactive species production and antioxidant or repair capacity, leading to oxidative modification of lipids, proteins, DNA, and RNA [75]. Redox effects of nucleotide interventions are therefore best evaluated through changes in oxidative stress biomarkers, endogenous antioxidant defenses, and mitochondrial function rather than assumed direct radical scavenging activity [48]. Most of the available evidence is preclinical. In H_2_O_2_-exposed human umbilical vein endothelial cells (HUVECs), a nucleotide-containing supplement reduced ROS and MDA, increased cell viability, and lowered selected cellular senescence markers [76]. Individual nucleotides produced different response patterns, indicating that the effects of a mixture cannot be attributed equally to all components. In H_2_O_2_-injured PC-12 cells, exogenous nucleotides similarly reduced ROS and MDA, increased SOD and GPx activities, decreased DNA damage and apoptosis, and increased mitochondrial membrane potential and ATP production [59]. Activation of the NAD^+^/SIRT1/PGC-1α pathway in this model points toward mitochondrial regulation rather than simple free radical scavenging [59]. Animal studies show a broadly similar pattern but also reveal strong model dependence. In SAMP8 mice, long-term supplementation was associated with changes in redox markers in brown adipose tissue and accompanied by AMPK/SIRT1-linked changes in thermogenic markers [58]. Xu et al. reported higher SOD and GPx activities together with changes in skeletal muscle energy enzymes and longer swimming time in male ICR mice [48]. Cardiac studies in aging SAMP8 mice also found higher antioxidant enzyme activities and changes in mitochondrial bioenergetic indices [47]. Related SAMP8 studies reported redox-associated changes in the skin, testis, and liver [49,77,78]. These studies extend the tissue range of the evidence, but their origin within the same broader aging nucleotide research program limits independent replication. Aquaculture models further illustrate dose dependence. In sterlet sturgeon (*Acipenser ruthenus*), 10-week supplementation altered SOD, CAT, GPx, total antioxidant capacity, and MDA at selected doses, whereas the highest dose did not consistently provide additional benefit [79]. Overall, the preclinical literature supports modulation of redox homeostasis and mitochondrial function in selected models. It does not establish universal antioxidant action or a clinically meaningful antioxidant effect in humans.

#### 4.3.2. Human Evidence and Translational Limitations

Human redox data remain limited. Gene-Morales et al. reported changes in selected redox, inflammatory, functional, and cognitive outcomes in adults aged 60–75 years, but participants were not selected for metabolic disease and the yeast-derived intervention contained additional nutrients [11]. TALENTs provided complementary aging-related metabolic evidence, including a reduction in HOMA-IR, but it was not designed as a redox trial or a study of clinically diagnosed insulin resistance [80]. These trials therefore provide preliminary translational evidence rather than proof of a clinically meaningful antioxidant or insulin-sensitizing effect. Full adult trial details are provided in Section 5.4. A neonatal piglet study offers a well-defined developmental redox comparator. Hu et al. studied 14 matched pairs of normal-birth-weight (NBW) and intrauterine-growth-restricted (IUGR) male piglets from postnatal day 7 to day 28. Within each birth weight stratum, piglets received control milk replacer or a nucleotide-supplemented replacer [81]. The nucleotide premix supplied 740.9 g of pure nucleotide mixture per 100 kg of milk replacer powder (7.409 g/kg), comprising 29.6 g AMP, 14.2 g CMP, 40.8 g GMP, 5.8 g IMP, and 650.5 g UMP per 100 kg of powder. Supplementation increased several plasma and hepatic antioxidant indices, including total antioxidant capacity (T-AOC), total superoxide dismutase (T-SOD), GPx, and the reduced-to-oxidized glutathione ratio (GSH/GSSG). It also reduced hepatic MDA and altered Nrf2-, PGC-1α-, and nuclear respiratory factor 1 (NRF1)-related expression. The precise composition is a strength, but the neonatal IUGR pig model and strongly UMP-dominant formulation limit extrapolation to adult insulin resistance. Table 4 provides a comparative overview of the principal redox-related intervention studies and their main limitations [81].

## 5. Dietary Nucleotides and Insulin Resistance

### 5.1. Carbohydrate Metabolism and Insulin Sensitivity

Insulin resistance is characterized by impaired insulin responsiveness in insulin-sensitive tissues and involves disturbances in insulin receptor substrate (IRS)/phosphoinositide 3-kinase (PI3K)/AKT signaling [10]. Song et al. established an insulin-resistant HepG2 model with 0.25 mM palmitic acid for 24 h and maintained palmitate for a further 24 h while adding a defined AMP:CMP:GMP:UMP mixture (16:41:19:24) at 50, 100, or 200 μmol/L. Metformin at 0.1 mg/mL served as the positive comparator, and cells received 1 μmol/L of insulin for 40 min before endpoint collection [10]. The nucleotide mixture increased glucose consumption and glycogen content and modulated hexokinase, PEPCK, G6Pase, IRS-1/AKT/FOXO1, AMPK, oxidative stress, inflammatory, and glucose transporter endpoints. This is the most direct mechanistic supplementation study relevant to hepatic insulin resistance in this review, but it remains a transformed hepatocellular cell line exposed to a short high-palmitate challenge. In 3-day-old weaned piglets, dietary nucleotide supplementation altered hepatic PI3K/AKT- and MAPK-related transcriptional pathways [73]. Separate hepatocyte studies establish the centrality and redundancy of PI3K-AKT signaling in insulin action but do not constitute nucleotide supplementation evidence [83].

Clinical evidence is limited to older adults who were not selected for metabolic disease. In the 19-week TALENTs trial, nucleotide supplementation reduced HOMA-IR compared with placebo (β = −0.45, 95% confidence interval (CI) −0.86 to −0.04, *p* = 0.033), a finding consistent with improved estimated insulin resistance rather than a direct measurement of insulin sensitivity [80]. A secondary analysis suggested that the response may vary according to fasting glucose polygenic risk [84], and a separate 2026 secondary analysis of the same TALENTs cohort reported genotype-dependent responses according to urate-related polygenic risk [85]. These analyses are exploratory and not independent clinical replications of the parent trial.

Studies of endogenous extracellular nucleotides provide additional mechanistic context. ENTPD5-mediated ATP-to-ADP hydrolysis contributes to hepatic glucose and lipid homeostasis and brown adipose tissue thermogenesis, whereas P2Y2 signaling may promote adipocyte differentiation and insulin resistance [66,71]. These pathways should be discussed separately from oral nucleotide supplementation because direct causal links between supplementation and local purinergic signaling have not been established.

### 5.2. Lipid Metabolism and β-Oxidation

Available evidence suggests that nucleotide-related interventions may influence hepatic lipid accumulation, AMPK–acetyl-CoA carboxylase (ACC) signaling, brown adipose tissue thermogenesis, and adipocyte function, but direct evidence for uniform stimulation of β-oxidation is limited [49,50,66,71]. In zebrafish, diets containing 0.1% nucleotides or 0.02% AMP reduced hepatic triglycerides and steatosis, and the effect depended on nucleoside uptake and AMPK–ACC activation [50]. In aged SAMP8 mice, long-term supplementation improved hepatic histopathology, antioxidant capacity, and lipid accumulation [49]. Conversely, in young piglets, nucleotide supplementation increased the expression of sterol regulatory element-binding protein 1c (SREBP1c) and fatty-acid-binding protein 1 (FABP1) while reducing adipose triglyceride lipase (ATGL) and carnitine palmitoyltransferase 1 alpha (CPT1α), indicating that effects on lipid handling vary with species, age, developmental stage, and metabolic context [73]. Nucleotide supplementation also increased thermogenesis-related markers in brown adipose tissue in aging models [58]. Separate studies of endogenous extracellular ATP signaling show that ENTPD5-dependent ATP-to-ADP hydrolysis can support hepatic metabolism and brown adipose tissue thermogenesis, whereas ATP-dependent P2X4/P2X7 activation in resident macrophages may promote inflammatory degeneration of thermogenic tissue [66,74]. These purinergic findings demonstrate context-dependent local signaling and should not be interpreted as direct effects of dietary nucleotide supplementation.

A substantial but previously underrepresented body of older direct dietary work comes from Wistar rat liver injury models. In the Granada thioacetamide program, female Wistar rats received 300 mg/L of thioacetamide in drinking water for 4 months. Torres et al. then compared 2-week recovery on a nucleotide-free diet with a diet containing 250 mg of nucleotides per 100 g diet (2.5 g/kg total) and reported improved hepatocyte morphometry and reduced collagen deposition [86,87]. Fontana et al. studied 54 female Wistar rats. After 4 months of thioacetamide exposure, recovery for 1 or 2 weeks on a diet containing 50 mg/100 g each of AMP, GMP, CMP, IMP, and UMP (2.5 g/kg total) corrected several plasma and liver microsomal fatty acid abnormalities [88]. Pérez et al. subsequently pair-fed thioacetamide-treated rats for 4 months with or without 3 g/kg each of AMP, IMP, CMP, GMP, and UMP (15 g/kg total) and reported reduced fibrosis with higher collagenase activity and lower prolyl-4-hydroxylase activity and TIMP-1 expression [89]. A later redox/protein synthesis paper from the same program contains an internal dose reporting discrepancy. The PubMed abstract states 3 g/kg of each monophosphate, whereas the published full-text Methods report 0.3 g/kg of each. The study is therefore included for mechanistic outcomes but not used for quantitative dose–response inference [90]. These publications are mechanistically informative but represent a related experimental cluster rather than independent replication. Related nutritional deprivation data reinforce that this older liver literature is not limited to chemically injured models. López-Navarro et al. fed adult rats a nucleotide-containing diet or the same diet without nucleotides for 21 days and observed smaller hepatocyte nuclear/nucleolar areas, chromatin condensation, reduced rough endoplasmic reticulum and ribosome abundance, and greater fat accumulation during nucleotide deprivation [91]. These findings support a role for exogenous nucleotide availability in hepatic structural maintenance, but the deprivation design does not establish that supplementation above normal nutritional sufficiency improves metabolic disease.

Individual pyrimidine exposures further illustrate why nucleotide classes and treatment duration should not be pooled. In 21 seven-day-old Duroc × Landrace × Yorkshire piglets (*n* = 7/group), Zhang et al. administered UMP at 0.119 g/mL × 4 mL/day (0.476 g/day) or uridine at 0.087 g/mL × 4 mL/day (0.348 g/day) orally for 10 days. Both interventions altered circulating lipids, hepatic fatty acid composition, and expression of liver X receptor alpha (LXRα), SREBP1c, fatty acid desaturase 2 (FADS2), elongation of very long-chain fatty acids protein 5 (ELOVL5), ATGL, hormone-sensitive lipase (HSL), CPT1α, and AKT-related endpoints [92]. Li et al. reported intestinal morphology, epithelial turnover, and pyrimidine metabolism outcomes from the same 21-piglet, three-group UMP/uridine experimental series [93]. The publication is therefore treated as a complementary report rather than an independent replication. Conversely, Urasaki et al. fed male C57BL/6J mice uridine at 400 mg/kg/day for 5 days or 16 weeks. Chronic 16-week exposure produced marked hepatic lipid accumulation, impaired glucose tolerance, reduced hepatic AKT signaling, and FOXO1-related gluconeogenic changes [94]. Uridine is a nucleoside, not a nucleotide, and these data should therefore be interpreted as a counterexample demonstrating exposure- and duration-specific biology rather than evidence against defined dietary nucleotide mixtures. Human neonatal studies add an important counterweight to the predominantly preclinical lipid literature. Gil et al. studied 84 term neonates during the first month of life. The cohort included 26 infants receiving human milk, 35 receiving standard formula, and 23 receiving the same formula supplemented with CMP, AMP, GMP, UMP, and IMP at concentrations intended to approximate human milk [95]. Plasma long-chain fatty acid patterns differed between standard- and nucleotide-supplemented formula groups, but the developmental model and human milk reference preclude inference about adult lipid lowering. DeLucchi et al. similarly evaluated 58 term infants at 30 days (human milk n = 20, standard formula n = 19, nucleotide-supplemented formula n = 19) and reported erythrocyte phospholipid polyunsaturated fatty acid (PUFA)/unsaturation patterns closer to the breast-fed reference in the supplemented group [96]. In preterm infants, Sánchez-Pozo et al. compared nucleotide-free and nucleotide-supplemented formula during the first month and observed transiently higher plasma lecithin–cholesterol acyltransferase (LCAT) activity and apolipoprotein A-IV concentrations [97]. In contrast, Axelsson et al. studied 40 preterm infants for 6 weeks (human milk n = 14, nucleotide formula n = 13, standard formula n = 13). The supplemented formula contained 18.2 mg/L of CMP, 7.0 mg/L of UMP, 6.4 mg/L of AMP, 3.0 mg/L of IMP, and 3.0 mg/L of GMP (37.6 mg/L total) [98]. It did not improve erythrocyte long-chain polyunsaturated fatty acids (LC-PUFAs) relative to standard formula and was associated with higher plasma triglycerides [98]. Together, these trials show that lipid-related effects are endpoint-, formulation-, and developmental-stage-specific rather than uniformly beneficial.

An additional prospective randomized study of 150 healthy preterm neonates found higher high-density lipoprotein cholesterol (HDL-C) and lower low-density lipoprotein cholesterol (LDL-C) after feeding a nucleotide-supplemented formula, whereas triglycerides, total cholesterol, and very-low-density lipoprotein cholesterol (VLDL-C) did not differ from unsupplemented formula [99]. The authors explicitly noted that clinical relevance was uncertain; together with the divergent neonatal studies summarized above, this further argues against extrapolating infant lipoprotein responses to adult lipid-lowering efficacy.

### 5.3. Mechanisms Linking Dietary Nucleotides with the Pathophysiology of Insulin Resistance

**Figure 3:**
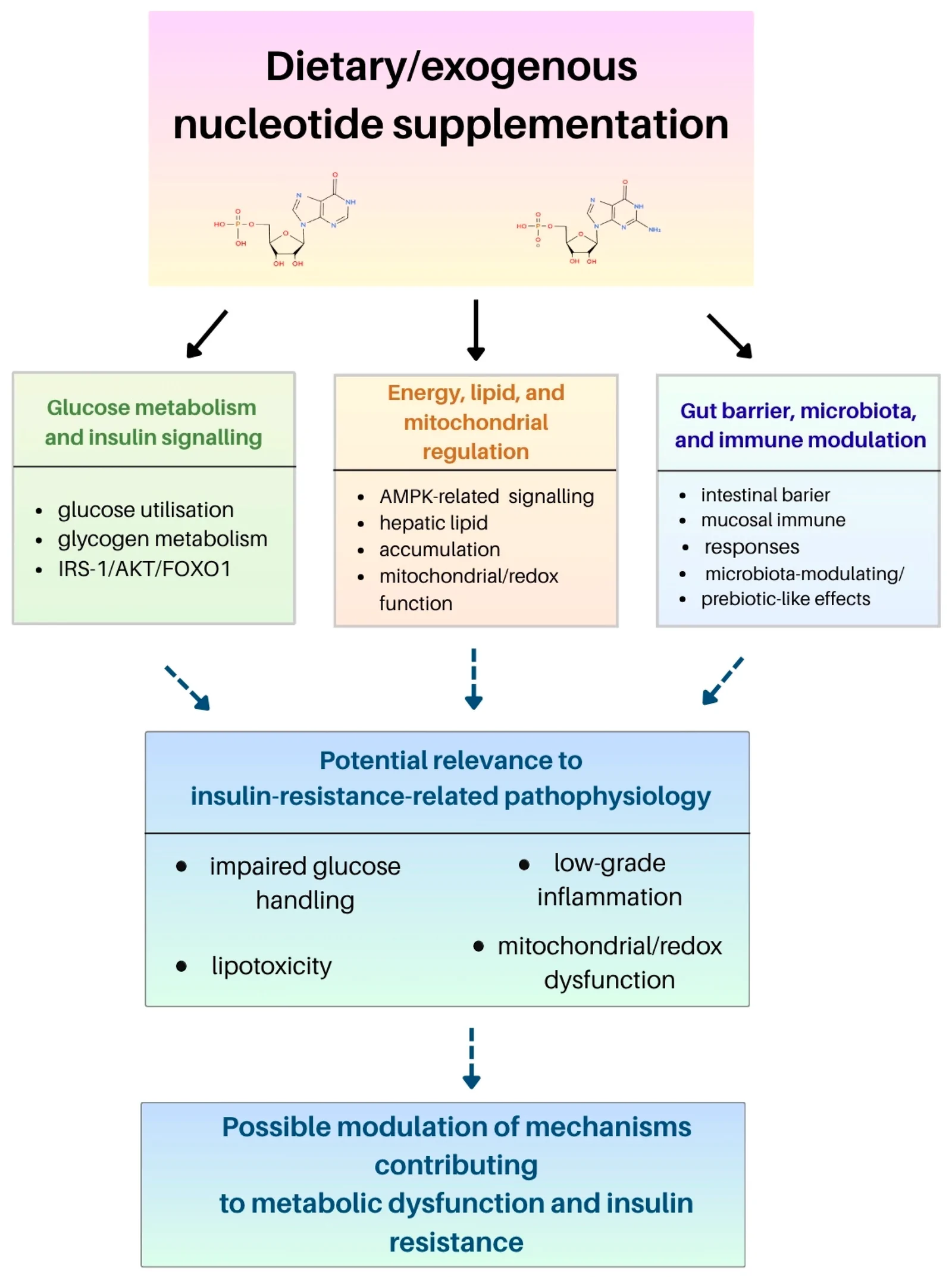
Proposed mechanistic links between dietary/exogenous nucleotide supplementation and pathways relevant to insulin resistance. Direct supplementation studies suggest potential effects on glucose metabolism and insulin signaling, energy, lipid, and mitochondrial regulation, as well as gut barrier function, microbiota, and immune modulation. Together, these processes may be relevant to insulin-resistance-related pathophysiology, including impaired glucose handling, lipotoxicity, low-grade inflammation, and mitochondrial/redox dysfunction. Solid arrows indicate relationships supported by direct supplementation evidence, whereas dashed arrows indicate potential, indirect, or still uncertain links. This figure summarizes mechanistic concepts and should not be interpreted as evidence of established clinical efficacy. Abbreviations: AKT, protein kinase B; AMPK, AMP-activated protein kinase; FOXO1, forkhead box protein O1; IRS-1, insulin receptor substrate 1. The figure was prepared using free online software available at https://www.canva.com/.

Overall, the evidence points to three linked domains relevant to insulin resistance, namely hepatic insulin and energy signaling, lipid and redox metabolism, and gut–immune regulation. Evidence remains predominantly preclinical, so these links should be interpreted as mechanistic hypotheses rather than established therapeutic effects. The strongest direct mechanistic evidence comes from palmitate-treated HepG2 cells, where exogenous nucleotides affected glucose utilization, glycogen content, IRS-1/AKT/FOXO1 signaling, and AMPK activity [10]. This supports a hepatic mechanism but not whole-body insulin sensitization. Animal data extend this pattern to lipid and bioenergetic regulation in zebrafish and aging SAMP8 mice, but the magnitude and direction of the effects vary by model and exposure [49,50]. Intestinal barrier function, the gut microbiota, and mucosal immune signaling may provide an indirect gut–liver route relevant to insulin resistance [4,5,6,36,100]. Evidence from food-derived nucleic acids or complex mixtures, however, cannot be assigned to isolated nucleotide supplements. Endogenous purinergic signaling remains contextual. ATP-dependent pathways involving P2X7, P2Y2, and ENTPD5 regulate glucose transport, adipocyte biology, hepatic metabolism, and thermogenesis, but oral supplementation has not been shown to reproduce these local effects [66,67,71]. Human evidence is discussed in Section 5.4. The available trials remain hypothesis-generating because they enrolled older adults rather than metabolically selected patients, and secondary TALENTs analyses are not independent replications [80,84,85]. Figure 3 summarizes this evidence hierarchy.

### 5.4. Human Intervention Evidence and Translational Limits

The available adult intervention evidence is confined largely to adults not selected for metabolic disease. Gene-Morales et al. randomized 69 physically independent adults aged 60–75 years for 10 weeks to a yeast-derived preparation (250 mg/day, >40% free nucleotides), a four-monophosphate formulation containing AMP, CMP, UMP, and GMP (150 mg/day), or placebo [11]. Both supplements were administered as one daily capsule. The yeast-derived preparation also contained amino acids, minerals, and B-group vitamins, so effects from that arm cannot be attributed solely to nucleotides. TALENTs randomized 121 adults aged 60–70 years in a 1:1 allocation for 19 weeks to 1.2 g/day of yeast-derived 5′-AMP:5′-CMP:5′-GMPNa2:5′-UMPNa2 in a 16:41:19:24 ratio or placebo [80]. HOMA-IR decreased (β = −0.45, 95% CI −0.86 to −0.04, *p* = 0.033), but this index is an estimate rather than clamp-derived insulin sensitivity. Two 2026 multi-omics reports by Fu et al. evaluated fasting-glucose-related and urate-related polygenic risk within the same TALENTs cohort [84,85]. Both are secondary analyses and not independent clinical replications. Coelho et al. randomized 20 healthy adults to a 7-day fully controlled eucaloric diet containing twice-daily nucleotide-depleted or high-nucleotide mycoprotein meals. The intervention increased serum uric acid but did not measurably alter insulin sensitivity indices, 24 h glycemia, or the plasma lipidome [101]. At present, no randomized trial has specifically tested defined nucleotide supplementation in patients selected for insulin resistance, type 2 diabetes, MASLD, or obesity-related inflammatory dysfunction.

Adult gastrointestinal evidence includes a small randomized double-blind placebo-controlled crossover trial in 37 participants with irritable bowel syndrome. IntestAidIB was administered as 500 mg capsules three times daily for 56 days per treatment period and produced modest improvements in some symptom scores, but the formulation contained yeast-derived nucleotides/RNA together with fructo-oligosaccharides, amino acids, and vitamins, precluding attribution of the effects to nucleotides alone [102]. The study did not assess insulin resistance or chronic metabolic disease.

Earlier adult trials addressed exercise stress physiology rather than chronic metabolic disease. McNaughton et al. randomized 14 moderately trained men (n = 7/group) to a nucleotide-containing supplement or placebo for 60 days. Before and after supplementation, participants completed 90 min of cycling at 60% of maximal oxygen uptake (VO_2_max) [103]. The product was a multi-ingredient yeast/RNA-derived nucleotide preparation rather than a chemically defined 5′-monophosphate intervention, and the reported signal concerned attenuation of post-exercise cortisol together with higher salivary immunoglobulin A (IgA). In a second 60-day trial, 30 moderately trained men were allocated to control, placebo, or nucleotide supplement groups (n = 10 each) and completed a 2 min maximal exercise test. Salivary cortisol and IgA responses changed, whereas lactate, lactate dehydrogenase (LDH), and creatine kinase did not [104]. These studies broaden the adult human evidence base but do not demonstrate improved insulin resistance. Human intestinal physiology has also been studied in preterm infants. Carver et al. evaluated 20 formula-fed preterm infants receiving an 80.6 mg/L nucleotide-containing formula or the same formula without added nucleotides, with 20 human-milk-fed preterm infants as a reference [105]. Superior mesenteric artery blood flow velocities were measured before feeding and at 30, 60, and 90 min afterward. The late postprandial response differed between nucleotide-positive and nucleotide-negative feeds. This acute neonatal vascular endpoint should not be extrapolated to adult systemic metabolic efficacy.

A later double-blind crossover study in 20 adults (10 men and 10 women) examined heavy resistance exercise and found lower immediate post-exercise cortisol and myeloperoxidase and lower creatine kinase at 24 h during nucleotide supplementation, without a treatment effect on leukocyte counts [106]. These findings extend exercise stress evidence across sexes but remain unrelated to chronic metabolic inflammation. In term infants, Carver et al. also found higher 90 min postprandial superior mesenteric artery blood flow velocity after four weeks of nucleotide-supplemented formula than after unsupplemented formula while emphasizing that clinical significance was unknown [107].

### 5.5. Dietary Nucleotides in the Context of an Anti-Inflammatory Diet

Anti-inflammatory diets are dietary patterns rather than effects of single nutrients. Mediterranean-type patterns rich in plant foods, fiber, unsaturated fatty acids, extra virgin olive oil, nuts, and polyphenols have the strongest evidence for reducing selected inflammatory biomarkers [8,9,108,109,110]. Dietary nucleotides are much less studied and should not be presented as equivalent to these established components.

Experimental studies suggest that nucleotide-containing interventions may support intestinal barrier integrity, mucosal immune regulation, redox homeostasis, and selected insulin-related pathways [10,36,37,100]. This evidence provides a rationale for testing defined nucleotide preparations as adjuncts within controlled dietary patterns, but no study has yet established that adding nucleotides improves the efficacy of an anti-inflammatory diet in humans. This concept should therefore be treated as a research hypothesis rather than a current dietary recommendation.

## 6. Effects of Dietary Nucleotides on the Immune System and Gut Microbiota

### 6.1. Effects on Immune Function

The immune system depends on the availability of purines and pyrimidines as precursors for DNA and RNA synthesis, processes required for the rapid clonal expansion of immune cells and for protein synthesis during immune activation [111]. Available experimental evidence suggests that dietary or exogenous nucleotides can modulate selected immune functions, especially mucosal immunity, lymphocyte proliferation, and cytokine responses, but the clinical relevance of these effects in adults remains insufficiently established [111]. In recent years, the immunological effects of nucleotides have been described particularly in the context of intestinal mucosal immunity, local cytokine regulation, and mechanisms involved in oral tolerance [36,37,112]. However, it should be emphasized that human studies remain limited, and most available evidence comes from animal models, in vitro systems, or specific nutritional contexts.

Foundational murine studies established that nucleotide deprivation and repletion can alter cellular and mucosal immunity, but these experiments should be interpreted in their nutritional deficiency context. Van Buren et al. showed suppressed helper/inducer T-lymphocyte-related responses during dietary nucleotide restriction [113]. Kulkarni et al. compared BALB/c mice maintained on chow, a nucleotide-free diet, or nucleotide-free diets repleted with RNA, adenine, or uracil and demonstrated impaired phagocytic function and markedly increased mortality after *Staphylococcus aureus* challenge in the nucleotide-free condition [114]. Carver et al. subsequently reported diet-dependent changes in murine natural killer cell activity and macrophage activation [115], while Navarro et al. found that a breast-milk-like nucleotide mixture restored selected antibody-forming-cell and ConA-driven lymphoproliferative responses in 20-day-old mice fed a nucleotide-free diet for 30 days [116]. These studies support the nutritional dependence of selected immune functions rather than a general pharmacological immunostimulatory effect. A particularly useful oral repletion study helps separate dietary exposure from parenteral nucleotide experiments. Jyonouchi and Sun fed C57BL/6 mice a nucleotide-free diet for 3 weeks and administered a defined mononucleotide mixture through daily gavage at 14 or 70 μmol/kg/day for the same period [117]. Both doses prevented the reduction in T-cell-dependent antibody production associated with the nucleotide-free diet. The model therefore supports oral nutritional repletion of a deficiency phenotype, but it does not demonstrate pharmacological immune enhancement in nucleotide-replete adults.

Later mechanistic mouse studies refined this intestinal immune phenotype. Nagafuchi et al. reported that dietary nucleotides enhanced antigen-specific type 1 helper T-cell (Th1) responses while suppressing antigen-specific immunoglobulin E (IgE) [118], increased the proportion of γδ T-cell receptor (TCRγδ)+ intestinal intraepithelial lymphocytes together with epithelial interleukin 7 (IL-7) production [119], and increased mucosal IgA responses with epithelial transforming growth factor beta (TGF-β) secretion in ovalbumin-specific T-cell-receptor transgenic mice [120]. Manzano et al. used a semi-purified diet supplemented with 3 g/kg each of AMP, CMP, GMP, and UMP (12 g/kg total) in weanling mice and sampled days 0, 4, 7, 12, and 18. Nucleotide supplementation accelerated developmental changes in Peyer patch, epithelial, and lamina propria lymphocyte phenotypes [121]. These are high-dose developmental models and do not establish adult metabolic efficacy.

Human immune intervention evidence also extends beyond infant formula and older adult trials. Carver et al. studied 37 term infants (9 breastfed, 13 receiving formula containing 33 mg/L of nucleotides, and 15 receiving standard formula) and reported higher natural killer cell activity and interleukin 2 (IL-2) production in the nucleotide formula group at 2 months [122]. Navarro et al. later compared preterm infants receiving standard formula or the same formula supplemented with CMP, AMP, UMP, GMP, and IMP to approximate human milk acid-soluble nucleotides. Immunoglobulin M (IgM) was higher across postnatal assessments and IgA at 3 months, whereas major lymphocyte subset differences were not observed [123]. In adults, Ostojic and Obrenovic randomized 38 healthy men aged 20–25 years (19/group) to sublingual nucleotides at 50 mg/day in three divided doses or placebo for 14 days and reported higher serum IgA, natural killer cell number/cytotoxicity, and attenuation of selected post-exercise salivary immune changes [124]. A second double-blind trial in 30 young physically active men used the same 50 mg/day sublingual dose for 14 days and reported longer time to exhaustion with higher IgA and natural killer cell cytotoxicity [125]. Riera et al. randomized 20 elite male taekwondo athletes (10/group) to Inmunactive^®^ 480 mg/day or placebo for 30 days and observed selected improvements in lymphocyte recovery and salivary/lymphocyte immune markers after exhaustive exercise in the cold [126]. Because the latter studies used exercise stress populations and, for Ostojic, a sublingual route specifically intended to bypass intestinal degradation and first-pass metabolism, they should not be treated as evidence for dietary treatment of insulin resistance.

The most consistent signal appears in conditions in which the intestinal barrier and the immune system operate under increased functional demand, such as early weaning, aging, intestinal stress, or the maintenance of tolerance to food antigens [36,37,100]. Clinical evidence in humans is more developed in infant nutrition than in adults. A systematic review and meta-analysis of ribonucleotide-supplemented infant formulas evaluated antibody responses to common pediatric vaccinations, lymphocyte subsets, natural killer cells, episodes of diarrhea, and acute respiratory infections, suggesting potential benefits in selected immune-related outcomes [29]. However, individual randomized trials have not uniformly demonstrated improvements in growth or immune biomarkers in healthy term infants [30]. Adult evidence is even more limited. One small study in athletes suggested that a nucleotide-based supplement may attenuate selected immune perturbations after strenuous exercise under cold conditions, but this stress model should not be extrapolated to chronic metabolic inflammation, insulin resistance, MASLD, or obesity [126].

A key primary infant trial that warrants separate recognition is that of Pickering et al. [127]. In this 12-month randomized, blinded, multisite study, 370 healthy term infants were enrolled, and 311 completed follow-up. The groups comprised 107 infants receiving control formula, 101 receiving formula fortified with nucleotides at 72 mg/L in a human-milk-like ratio, and 103 in the human milk/reference group. The nucleotide formula group showed higher selected antibody responses, including *Haemophilus influenzae* type b and diphtheria responses at specific time points, but effects were not uniform across all vaccines. This trial reinforces that the most developed human immune evidence concerns infant formula rather than adult metabolic inflammation.

Older controlled intestinal repair experiments provide direct evidence for condition-dependent effects. In 21-day-old rats with lactose-induced chronic diarrhea, Núñez et al. used 36 animals per initial dietary condition. After 15 days, recovery diets were given for 4 weeks with or without 50 mg/100 g each of AMP, GMP, CMP, UMP, and IMP (2.5 g/kg total). Nucleotide supplementation increased intestinal DNA and disaccharidase activities, particularly in the jejunum and ileum, during recovery [128]. Bueno et al. used a related weanling rat diarrhea model and the same five-monophosphate dose (50 mg/100 g each) during 4 weeks of recovery. Villus/crypt architecture, goblet cells, intraepithelial lymphocytes, and mitochondrial ultrastructure were closer to controls in the nucleotide group [129]. In 17-month-old rats deprived of food for 5 days, refeeding for 3 or 6 days with nucleotide supplementation accelerated restoration of jejunal and ileal mucosal growth and differentiation markers relative to a nucleotide-free diet [130]. In a separate adult Wistar model, dietary nucleotide deprivation produced time-dependent reductions in brush border maturation enzymes, especially toward the villus tip, supporting a role in enterocyte maturation rather than a generic anti-inflammatory action [131]. Conversely, in male Sprague–Dawley rats given 40 g/L of dextran sulfate sodium (DSS) for 3 days after 2 days of nucleotide-free or nucleotide-supplemented prefeeding, nucleotide supplementation did not improve histology and was associated with higher colonic myeloperoxidase activity and more persistent interleukin 1 beta (IL-1β), indicating aggravation rather than protection in this colitis model [132].

Dinardo et al. evaluated oral yeast-derived nucleotide supplementation from birth to day 25 in Holstein Friesian calves [112]. The study assessed oxidative stress markers, immune cell function, serum biochemistry, and intestinal mucosal development. Supplemented calves showed lower intracellular ROS in peripheral blood mononuclear cells and higher activities of selected endogenous antioxidant enzymes in the plasma and liver, while serum IgG did not change significantly [112]. The pattern is more consistent with redox and mucosal immune modulation than with broad humoral immune stimulation.

Dose–response immune evidence is also available in adult animal models. Xu et al. allocated female BALB/c mice to a standard control, nucleotide-free control, or four nucleotide-supplemented diets spanning 0.0025% to 0.64%. The 0.04% group most closely restored several T-cell, macrophage, NK-cell, and cytokine measures toward control values [133]. These findings support immune modulation but also show that higher nucleotide doses were not uniformly superior.

Across these animal studies, immune effects vary with model and dose rather than showing a uniform stimulatory pattern.

The most mechanistically detailed evidence comes from the 2024 study by Yang et al., which investigated a broad spectrum of food-derived nucleic acids rather than traditional mixtures of free nucleotides [100]. In a mouse model, the authors showed that dietary RNA and DNA can activate mitochondrial antiviral-signaling protein (MAVS)/stimulator of interferon genes (STING)–TANK-binding kinase 1 (TBK1)-dependent innate sensing pathways in the small intestine, leading to interleukin 15 (IL-15) induction and maintenance of natural intraepithelial lymphocytes (IELs) and support of oral tolerance to dietary antigens [100]. A further consequence of this pathway was transforming growth factor beta 1 (TGF-β1) production by natural IELs and activation of cluster of differentiation 103 (CD103)+ dendritic cells, which promoted the generation of antigen-specific regulatory T cells. That study is particularly important because it suggests that nucleic-acid-derived dietary components may directly contribute to intestinal immune homeostasis and oral tolerance, rather than acting only indirectly through improvement of the epithelial barrier [100]. However, because this evidence comes from mice and involves food-derived nucleic acids broadly, it should not be interpreted as direct clinical proof of the efficacy of isolated nucleotide supplements in humans.

In a model of intestinal aging, the effects of exogenous nucleotides showed a somewhat different profile. In SAMP8 mice, long-term nucleotide supplementation improved colonic morphology, increased trefoil factor 3 (TFF3) levels and telomerase activity, and elevated IgA concentrations in colonic tissue [37]. At the same time, supplementation reduced monocyte chemoattractant protein 1 (MCP-1) levels, and in some groups a tendency towards lower levels of other inflammatory markers was observed. The authors also reported activation of the p38 signaling pathway, whereas higher nucleotide doses showed a tendency to increase Nrf2 expression. In this aging model, nucleotides may therefore support local mucosal immunity and partially reduce chronic low-grade intestinal inflammation [37]. Nevertheless, these results remain preclinical and should be interpreted as mechanistic evidence rather than direct evidence of clinical efficacy in humans. Additional data come from a study in kittens in which a diet supplemented with nucleotides and oligosaccharides was evaluated [134]. Animals receiving the test diet showed reduced expression of selected circulating microRNAs (miRNAs), including miR-1-3p, miR-133a-3p, miR-206-3p, and miR-383-5p. Bioinformatic analysis suggested that potential targets of these miRNAs include genes and pathways involved in immune regulation. However, this finding should not be regarded as direct evidence of improved immune function because the intervention contained multiple bioactive components, the sample size was small, and the endpoints were molecular biomarkers rather than functional immune outcomes. Nevertheless, these data suggest that the nucleotide-containing component of complex diets may participate in the regulation of molecular factors involved in early immune development [134].

### 6.2. Role of Dietary Nucleotides in the Modulation of Gut Microbiota

The gastrointestinal tract harbors a complex microbial ecosystem that functions as a biochemical interface between diet and host physiology [135]. Evidence that nucleotide supplementation modifies this ecosystem is derived predominantly from infant, animal, and in vitro studies. There is currently no convincing adult human intervention evidence showing that nucleotide supplementation improves metabolic outcomes through microbiota modification. Accordingly, taxonomic shifts should not be equated with microbial function or host benefit without corresponding functional, metabolomic, or clinical endpoints. Not all intestinal inflammatory models support a beneficial direction of effect. In Sprague–Dawley rats maintained for 4 weeks on a nucleoside/nucleotide-free 20% casein diet or the same diet supplemented with a 0.5% nucleoside–nucleotide mixture, Adjei et al. induced trinitrobenzene sulphonic acid/ethanol colitis after 2 weeks [136]. The supplemented animals developed greater epithelial injury and inflammatory damage than nucleotide-deprived animals. This negative result is model-specific, but it is important because it demonstrates that nucleotide availability can amplify rather than suppress selected inflammatory responses depending on context.

Cell culture and developmental animal evidence indicates that nucleotide availability can influence intestinal epithelial growth, maturation, and immune-related function under conditions of high proliferative demand [35,137]. A particularly well-defined recent model used 96 piglets weaned at 3 days of age (48 females and 48 males, six replicates per treatment, and eight piglets per replicate) receiving basal artificial milk with or without 0.035% nucleotides in an equimolar/equal-ratio CMP:UMP:AMP:GMP:IMP mixture through day 35 [36]. Supplementation increased ileal villus height and villus height/crypt depth ratio, altered zonula occludens 1 (ZO-1), mucin, and cytokine/nuclear factor kappa B (NF-κB)-related endpoints, increased colonic short-chain fatty acids, and shifted the Firmicutes/Bacteroidota ratio and selected genera. Because inflammatory cytokines also increased, the response is better described as immune and barrier modulation than uniformly anti-inflammatory. These developmental data should not be interpreted as proof of microbiota-mediated metabolic benefit in adults.

Singhal et al. conducted a randomized controlled trial in formula-fed infants assigned to nucleotide-supplemented formula (31 mg/L, n = 35) or standard formula without added nucleotides (n = 37) with a breastfed reference group (n = 44) [31]. Nucleotide supplementation reduced the fecal Bacteroides–Porphyromonas–Prevotella-to-Bifidobacterium ratio relative to control formula, indicating a compositional shift toward the profile observed in the breastfed reference group. Because the study assessed selected microbial groups rather than whole-community function, this finding should be described as microbiota modulation rather than proof of a health benefit. In an in vitro infant fecal fermentation model, Qu et al. observed dose-dependent changes in selected genera, while experiments in neonatal rats suggested effects on microbiota–gut–brain development [138]. These results support early-life microbiota-modulating or prebiotic-like activity but do not establish adult metabolic benefit. They also show that higher nucleotide exposure can produce a different microbial profile from lower exposure.

Human microbiota findings are not directionally consistent. In an earlier study, Balmer et al. compared 32 infants receiving nucleotide-supplemented formula, 33 receiving unsupplemented formula, and 21 breastfed infants; at two weeks, nucleotide supplementation was associated with more frequent Escherichia coli colonization and fewer bifidobacteria and did not shift the fecal flora toward the breastfed pattern [139]. This negative human result is important when interpreting the later compositional shifts reported by Singhal et al. A separate 2025 neonatal rat study by Qu et al. found selective delivery of dietary nucleotide-derived compounds, particularly uridine- and uracil-related species, to the colon together with dose-dependent changes in gut development, hepatic metabolism, microbiota, and fecal metabolites [140]. Because this work is developmental and overlaps conceptually with the same group’s microbiota–gut–brain program [138], it should be viewed as an extension of that evidence cluster rather than independent adult metabolic validation.

Mechanistically, microbiota changes need not mediate every intestinal immune effect. In germ-free zebrafish, Guo et al. showed that 0.1% dietary nucleotides enhanced selected barrier and innate immune markers and disease resistance, whereas transfer of nucleotide-associated microbiota did not reproduce the immune phenotype [141]. This supports a direct nucleotide effect in that model, but its aquaculture and germ-free context sharply limits translation to human metabolic disease.

Several controlled piglet studies provide useful dose and formulation information that was underrepresented in the earlier manuscript. Martinez-Puig et al. performed two early-weaning experiments with a sow-milk-inspired product (Nucleoforce Piglets^®^). In the first, three groups of six weaned piglets received 0, 1000, or 2000 ppm for 7 days alongside six unweaned controls. Villus height increased from 275 μm in unsupplemented weaned controls to 351 and 378 μm at 1000 and 2000 ppm. In a second 14-day trial of 384 piglets, 0, 750, or 1000 ppm did not change average daily gain or feed intake but reduced the proportion requiring antibiotic treatment for diarrhea from 15.63% to 3.13% and 1.56%, respectively [142]. Li et al. then studied 140 28-day-old weanling pigs for 42 days with a hydrolyzed *Kluyveromyces fragilis* yeast extract enriched in free nucleotides (Rovimax™ NX) at 0, 150, 220, or 275 mg/kg diet. A parallel 28-pig challenge experiment used the same diets and an oral *E. coli* K88 challenge (1.5 mL of 10^10 CFU/mL on day 14) [143]. Supplemented pigs showed improved growth/digestibility, and challenged animals showed higher IgA, IgM, and insulin-like growth factor 1 (IGF-1), with lower cortisol and tumor necrosis factor alpha (TNF-α), fecal *E. coli*, and fecal scores [143]. The yeast-derived composition and infection model limit direct extrapolation to purified nucleotides in humans.

Jang and Kim randomized 50 newly weaned pigs (19 days old, 25 barrows and 25 gilts, initial body mass 4.76 ± 0.42 kg) to 0, 50, 150, 250, or 500 mg/kg of dietary nucleotides for 21 days [144]. Feed intake and growth responses were most evident at 50–150 mg/kg, while villus/crypt and inflammatory endpoints showed non-linear dose responses, reinforcing that the highest dose is not necessarily optimal. Perricone et al. randomized 28 male post-weaning piglets to oral nucleotide mixture at 0.8 g/head/day in 2.1 mL of water or saline for 28 days [145]. Body weight, average daily gain, and feed intake increased, whereas circulating IgA/IgG and ileal Peyer patch interleukin 1 alpha (IL-1α), IL-1β, interleukin 6 (IL-6), interleukin 10 (IL-10), TNF-α, Toll-like receptor 2 (TLR2), Toll-like receptor 4 (TLR4), and peroxisome proliferator-activated receptor gamma (PPARγ) expression were not clearly altered [145]. These contrasting endpoints argue against treating growth improvement as synonymous with systemic immune activation.

Recent studies also provide important neutral and nutrient-context-dependent evidence. Correia et al. randomized 180 21-day-old piglets (5.17 ± 0.57 kg, nine pens of five pigs/treatment) for 24 days to control, purified nucleotide, autolyzed yeast, or sodium butyrate diets [146]. The nucleotide product contained 15% free nucleotides and was supplied to provide 100 mg of free nucleotides/kg diet in phase 1 and 75 mg/kg in phase 2. Unlike the yeast and butyrate arms, the purified nucleotide arm did not improve growth or the evaluated gut health endpoints. Lawal et al. studied 210 piglets for 35 days after weaning using a 24% crude protein positive control, a 16% low-protein control, or the low-protein diet plus Nucleosaf 600 at 1, 3, or 9 g/kg [147]. Nucleotide supplementation restored overall average daily gain toward the high-protein control, reduced blood urea nitrogen, increased nitrogen digestibility, and at 9 g/kg reduced post-weaning diarrhea. IGF-1 increased linearly with dose. In a subsequent 42-day trial of 160 pigs, Lawal et al. compared normal protein and low-protein diets with or without branched-chain amino acids (BCAAs) and 9 g/kg of Nucleosaf 600 [148]. The nucleotide preparation provided additional benefits particularly after BCAA supplementation, including nitrogen digestibility and partial recovery of growth/IGF-1, illustrating that background amino acid adequacy can materially modify the observed response.

Animal studies provide additional but context-dependent evidence. Wu et al. evaluated 60 one-day-old specific-pathogen-free chickens receiving 0%, 0.1%, 0.3%, or 0.5% yeast-derived nucleotides [149]. Supplementation altered bacterial diversity and increased *Lactobacillus* abundance while also changing intestinal morphology and barrier-related gene expression. Genus-level enrichment of *Lactobacillus* should not be equated with a confirmed probiotic or health effect, and the use of a yeast-derived preparation prevents attribution of all observations to a single purified nucleotide. These data are best interpreted as a coupled microbiota–barrier response in a juvenile animal model [149].

Maternal exposure should be separated from direct supplementation of the offspring. Gao et al. assigned 64 late-gestation Large White × Landrace sows (32/group) to a basal diet or the same diet plus 4 g/kg of yeast-based nucleotide preparation from gestation day 85 to lactation day 20 [150]. Maternal supplementation increased litter weaning weight, reduced piglet diarrhea, increased neonatal ileal villus development and secretory IgA, and altered barrier/immune endpoints. Because the preparation contained yeast-derived material (including 10.37% total nucleic acid) and exposure occurred through the dam, the study is supportive developmental evidence but not equivalent to a purified oral nucleotide intervention in adult humans.

Similar observations were reported by Valini et al. in a study conducted in weaned piglets [151]. In this experiment, 96 piglets aged 21 days were randomly assigned to three groups. They received a control diet, a diet supplemented with purified nucleotides, or an antibiotic-containing diet. The intervention lasted 14 days. The authors assessed microbiota composition in different intestinal segments as well as morphological and immunological parameters of the intestinal mucosa [151]. The results suggested that nucleotide supplementation exerted segment-specific effects on intestinal microbiota. In the ileum, an increase in total bacterial counts, including Enterobacteriaceae, was observed. This finding should be interpreted as a region-specific microbial shift rather than as unequivocal evidence of beneficial microbial diversity because Enterobacteriaceae may include both commensal and potentially pathogenic taxa. In contrast, in the colon, nucleotide supplementation reduced total bacterial counts, which the authors related to improved mucosal development and reduced availability of fermentable substrates. No significant effect on lactic acid bacteria was observed [151].

Another relevant aspect of nucleotide action in this model was the modulation of local intestinal immune responses. Nucleotide supplementation increased the number of B cells in the jejunum, whereas their number decreased in the ileum, suggesting region-specific remodeling of mucosal immunity rather than a uniform stimulatory effect. These changes were accompanied by a reduction in Paneth-cell-related parameters. Because Paneth cells are involved in the production of antimicrobial peptides, including defensins, this observation may indicate modulation of local antimicrobial mechanisms within the intestinal mucosa [151]. In addition, nucleotide supplementation influenced intestinal mucosal development and remodeling by increasing enterocyte proliferation and villus height. The observed changes in epithelial turnover, reflected by the balance between proliferative and apoptotic processes, suggest an effect on mucosal renewal [151]. Such structural and immunological changes may secondarily influence gut microbiota composition by modifying the intestinal barrier and local ecological conditions.

Two related Wistar rat publications from Cai et al. evaluated dietary nucleotide supplementation in alcohol-induced liver injury [152,153]. Male Wistar rats received a basal diet with 50% (*v*/*v*) alcohol exposure and nucleotide supplementation at 0.04% or 0.16% of the diet (0.4 or 1.6 g/kg). Outcomes included liver enzymes and histology, oxidative stress indices, inflammatory signaling, plasma lipopolysaccharide, cecal microbiota, and liver metabolomics through ultra-performance liquid chromatography–quadrupole time-of-flight mass spectrometry [152,153]. Nucleotide supplementation attenuated several alcohol-associated hepatic and metabolic abnormalities and altered microbiota/metabolite profiles. Because the two papers represent closely related experimental work from the same group, they are interpreted as one evidence cluster rather than independent replication. Alcoholic liver injury also differs mechanistically from insulin resistance and MASLD.

More recently, Han et al. randomized 360 swamp eels (initial body mass 10.07 ± 0.92 g) to six diets in triplicate for 8 weeks, containing 0, 0.25, 0.50, 0.75, 1.0, or 2.0 g/kg of a defined AMP/GMP/UMP/CMP mixture [154]. The estimated optimum for growth was approximately 0.76 g/kg, and dose-dependent changes in redox indices, intestinal morphology, gut microbiota, and hepatic/serum biochemical endpoints were reported. The study is useful because host-level outcomes accompany the microbiota data and because the dose–response was not monotonic at every endpoint, but it remains aquaculture evidence with low direct external validity for adult human metabolic disease.

Taken together, available data suggest that dietary nucleotides may influence gut microbiota through several interconnected mechanisms, including direct effects on selected microbial populations, support of intestinal epithelial renewal, modulation of mucosal barrier function, and regulation of local immune responses. In animal nutrition, the combined evidence indicates changes in microbiota, barrier, and mucosal endpoints under conditions in which antibiotic growth promoters are restricted [151]. However, this interpretation should not be extrapolated to human antibiotic therapy. According to the current International Scientific Association for Probiotics and Prebiotics (ISAPP) consensus definition, a prebiotic is a substrate that is selectively utilized by host microorganisms and confers a health benefit [155]. Therefore, dietary nucleotides should currently be described as compounds with potential prebiotic-like or microbiota-modulating activity rather than established prebiotics, particularly in adults with insulin resistance, MASLD, or obesity. Overall, the microbiota evidence remains predominantly developmental or preclinical and should be considered hypothesis-generating rather than directly applicable to adults with insulin resistance.

## 7. Safety, Limitations, and Future Perspectives

Safety depends on dose, source, duration of intake, and individual purine and urate handling. The fortification levels and supplemental doses evaluated in available human intervention studies have generally been well-tolerated in the populations studied, but the evidence base is dominated by infant formula studies, animal toxicology, and short adult interventions. Direct human evidence for an increase in uric acid comes from the randomized study by Coelho et al., in which seven days of high dietary nucleotide intake increased circulating uric acid from 295 ± 17 to 472 ± 29 μmol/L by day 6 without measurable deterioration of insulin sensitivity, glycemic regulation, or plasma lipid composition [101]. The infant immune study by Schaller et al. [156] and the multigeneration rat study [157] should not be cited as evidence that high purine intake causes hyperuricemia. They address infant immune responses and preclinical reproductive/developmental safety, respectively. Preclinical studies reported no major adverse effects at dietary nucleotide levels up to 1.28% in maternal rats and offspring [157] or up to 0.64% in a lifetime Sprague–Dawley study [158]. Infant studies provide additional reassurance regarding growth and immune outcomes at formula fortification levels [27,30,156]. Because uric acid is the final product of purine degradation in humans and urate elimination depends on renal and intestinal pathways, including ATP-binding cassette subfamily G member 2 (ABCG2)-mediated transport [159,160], high-dose supplementation warrants particular caution in individuals with pre-existing hyperuricemia, gout, chronic kidney disease, or impaired urate handling. Such populations were not specifically tested in the available nucleotide intervention trials. Longer-duration safety evidence is sparse and often embedded in specialized animal models. Yokoyama et al. fed male B6C3F1 mice a purified nucleotide-free diet supplemented with a nucleoside/nucleotide mixture at 0%, 0.5%, or 2.5%, beginning 1 week before and continuing for 13 months after californium-252 neutron irradiation [161]. Supplementation did not increase radiation-associated tumor incidence and was associated with fewer selected non-neoplastic lesions, including amyloidosis at 0.5%. The long duration is informative, but irradiation and the mixed nucleoside/nucleotide formulation substantially limit direct inference for routine human supplementation.

Overall, the fortification levels and supplemental doses evaluated in available human intervention studies appear generally well-tolerated, but long-term safety has not been established in metabolically vulnerable groups. Future trials should monitor serum uric acid, renal function, and adverse events, particularly during prolonged or high-dose exposure. These safety limitations should be considered separately from the mechanistic evidence discussed above.

The evidence base remains predominantly preclinical, heterogeneous in formulation and dose, and sparse in well-defined metabolic populations. Purified monophosphates, defined mixtures, yeast-derived products, nucleosides or nucleobases, dietary nucleic acids, and purine-rich foods should not be treated as interchangeable exposures. Several older hepatic and intestinal papers came from related University of Granada Wistar rat programs, while many later positive aging and redox studies came from the same Peking University/SAMP8 research network. These clusters provide multiple mechanistic endpoints but not independent replication across laboratories. Exposure ranges widely from infant formula fortification in the tens of mg/L to animal diets containing approximately 0.035–1% nucleotide mixtures and, in some liver injury studies, gram per kilogram five-monophosphate mixtures. Individual nucleoside studies use different dosing paradigms again. Because source studies report exposure in heterogeneous units (e.g., mg/L, mg/kg diet, percentage of diet, mg/head/day, or mg/kg body weight/day), doses are retained in the original study units unless a direct conversion is possible from reported data; conversions requiring assumptions about food intake, body mass, or product composition are not used. HepG2 cells, zebrafish, juvenile piglets, sturgeon, swamp eel, and aging mice therefore have limited external validity for adult human insulin resistance. Negative or divergent findings, including worsened DSS colitis and chronic uridine-associated steatosis/glucose intolerance, also show that benefit cannot be assumed across tissues, exposures, or durations [94,132]. Importantly, the available evidence also includes null and adverse findings. Some interventions failed to improve metabolic, lipid, immune, or intestinal outcomes, whereas others were associated with increased uric acid, worsened experimental colitis, or adverse metabolic effects during prolonged uridine exposure. Thus, the current literature does not support a uniformly beneficial effect of nucleotide-related interventions. Overall, intestinal and immunomodulatory evidence is broader than evidence for clinically meaningful metabolic improvement in insulin-resistant adults. Dose–response relationships remain incompletely defined, and higher doses are not consistently more favorable.

Future studies should therefore use chemically defined nucleotide preparations with standardized qualitative and quantitative compositions. They should be conducted in well-characterized populations with insulin resistance, visceral obesity, MASLD, or type 2 diabetes and should include clinically relevant endpoints such as HOMA-IR, fasting insulin, oral glucose tolerance, glycated hemoglobin (HbA1c), liver fat content, inflammatory markers, oxidative stress markers, gut barrier parameters, microbiota composition, uric acid concentration, and renal function.

## 8. Conclusions

In conclusion, dietary and exogenous nucleotides are potential modulators of metabolic and inflammatory pathways, but the current evidence is not sufficient to recommend them as independent therapeutic agents. Their implications for insulin resistance should therefore be regarded as biological plausibility and a translational research hypothesis rather than demonstrated clinical benefit. Their future role should be evaluated as adjunctive nutritional strategies using chemically defined preparations and clinically relevant endpoints in well-characterized metabolic populations.

## Figures and Tables

**Table 1:** Representative dietary nucleotide compounds and their nutritional sources.

Nucleotide	Full Name	Natural Dietary Sources	Fortified Products/Preparations	References
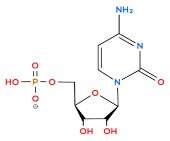 CMP	Cytidine 5′-monophosphate	Human milk, cow’s milk	Infant formulas and nutritional preparations for adults and children	[23,24,25,26,32]
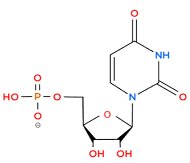 UMP	Uridine 5′-monophosphate	Human milk, dairy products	Infant formulas, nutritional preparations for adults and children, and foods for special medical purposes	[23,24,25,26,27,32,33]
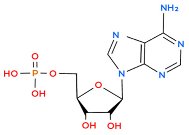 AMP	Adenosine 5′-monophosphate	Human milk, foods of animal origin	Infant formulas and nutritional preparations for adults and children	[18,20,23,24,25,26,32]
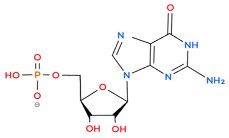 GMP	Guanosine 5′-monophosphate	Human milk, mushrooms, foods of animal origin	Infant formulas and nutritional preparations for adults and children	[18,20,21,23,24,25,26,32,34]
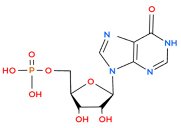 IMP	Inosine 5′-monophosphate	Meat, fish, products of animal origin	Infant formulas and nutritional preparations for adults and children	[19,20,23,32,34]

This table summarizes selected 5′-monophosphate nucleotides. Dietary nucleic acids, nucleosides, nucleobases, and total purine load are discussed separately because they do not directly correspond to free nucleotide intake. The molecular structures were prepared using free online software available at https://app.molview.com/.

**Table 3:** Contextual evidence linking extracellular purinergic signaling to metabolic regulation. None of the listed studies directly tests oral dietary nucleotide supplementation; the evidence is included to define tissue-specific mechanisms and should not be interpreted as supplementation efficacy.

Regulatory Pathway	Model/Design Context	Nucleotide/Metabolite or Manipulation	Key Molecular Mediators	Main Finding/Translational Limitation	References
Extracellular ATP hydrolysis at the enterocyte surface	Caco-2 cells; extracellular ATP hydrolysis in vitro	ATP → ADP/AMP/adenosine	NTPDase1/2, neutral ecto-phosphatase, ecto-5′-nucleotidase	Defines epithelial extracellular ATP metabolism; contextual evidence only.	[45]
Vesicular ATP release from hepatocytes	Mouse hepatocytes and Vnut−/− vs. wild-type mice; normal diet ≤ 28 weeks or HFD ≤ 48 weeks	Endogenous ATP release	VNUT, P2Y signaling	VNUT-dependent ATP release: ↑ triglyceride secretion and steatohepatitis-like pathology; pathophysiological context only.	[64]
ATP hydrolysis to ADP in the liver	Mouse genetic/mechanistic liver model	Extracellular ATP → ADP	ENTPD5	ENTPD5-dependent nucleotide metabolism altered hepatic glucose/lipid homeostasis and BAT thermogenesis; genetic context only.	[66]
ATP-dependent regulation of intestinal glucose transport	P2rx7−/− mice and enterocytes	Endogenous extracellular ATP	P2X7, GLUT2	P2X7 signaling: ↓ enterocyte glucose transport; receptor loss model, not dietary intervention.	[67]
ATP and glucose uptake in skeletal muscle	Primary myotubes/muscle fibers; electrical stimulation or exogenous ATP	Released or experimentally added ATP	PI3Kγ, AKT, AS160, GLUT4	ATP: ↑ GLUT4 translocation and glucose uptake, including under insulin-resistant conditions; acute local signaling.	[68]
Hydrolysis of extracellular nucleotides and energy expenditure	Global Entpd3 knockout vs. wild-type mice on HFD	ATP/ADP hydrolysis altered genetically	NTPDase3	NTPDase3 deletion: ↑ basal energy metabolism; ↓ diet-induced obesity; genetic model only.	[69]
ATP/UTP, adipogenesis, and insulin resistance	Human primary visceral preadipocytes/adipocytes; receptor agonism/antagonism ± inflammatory stimulation	ATP/UTP receptor signaling	P2Y2, AKT, GLUT4	P2Y2 signaling: ↑ terminal adipogenesis; ↓ AKT/GLUT4 signaling; human cells, not oral intervention.	[71]
Purine metabolism and thermogenesis	Mouse and human brown adipocyte systems; inosine/ENT1 manipulation	Inosine	ENT1, cAMP/PKA	Extracellular inosine: ↑ thermogenic activity; nucleoside evidence, not nucleotide supplementation.	[72]
Hepatic extracellular ATP and adenosine dynamics in steatotic liver disease	Zebrafish alcohol-associated liver disease/MASLD models; hepatocyte-specific GRAB sensors	Endogenous ATP/adenosine	Extracellular purine signaling	Disease-specific extracellular ATP/adenosine dynamics visualized in vivo; contextual disease mechanism only.	[65]
P2Y2 signaling in diet-induced obesity	HFD mouse model; P2Y2 pathway manipulation	ATP/UTP receptor signaling	P2Y2	P2Y2 signaling: ↑ obesity and adipose inflammation; receptor model, not oral intervention.	[70]
ATP-dependent adipocyte–macrophage crosstalk in BAT	Mouse BAT under thermoneutrality/overnutrition; myeloid P2X4/P2X7 manipulation	Adipocyte-derived ATP	P2X4, P2X7	Adipocyte-derived ATP: ↑ BAT-resident macrophage activation and BAT degeneration; paracrine context only.	[74]

Abbreviations: ADP, adenosine diphosphate; AKT, protein kinase B; AMP, adenosine 5′-monophosphate; AS160, AKT substrate of 160 kDa; ATP, adenosine triphosphate; BAT, brown adipose tissue; cAMP, cyclic adenosine monophosphate; ENT1, equilibrative nucleoside transporter 1; ENTPD5, ectonucleoside triphosphate diphosphohydrolase 5; GLUT2, glucose transporter type 2; GLUT4, glucose transporter type 4; GRAB, G-protein-coupled receptor-activation-based; HFD, high-fat diet; NTPDase1, ectonucleoside triphosphate diphosphohydrolase 1; NTPDase2, ectonucleoside triphosphate diphosphohydrolase 2; NTPDase3, ectonucleoside triphosphate diphosphohydrolase 3; P1P2X4, purinergic receptor P2X4; P2X7, purinergic receptor P2X7; P2Y2, purinergic receptor P2Y2; PI3Kγ, phosphoinositide 3-kinase gamma; PKA, protein kinase A; UTP, uridine triphosphate; VNUT, vesicular nucleotide transporter. Symbols: ↑, increase; ↓, decrease.

**Table 4:** Redox-related effects of exogenous or dietary nucleotide interventions, stratified by evidence level, formulation, dose/design information, and major interpretive limitations. Human randomized controlled trials (RCTs) are labeled explicitly to prevent equivalence with cellular or animal evidence. Doses are reported in the units used by the source studies; cross-study conversion to mg/kg/day was not performed when this would require assumptions about food intake, body mass, or product composition.

Evidence Level/Model	Intervention/Formulation and Design	Evaluated Endpoints	Main Finding/Interpretive Limitation	References
Cellular: HUVEC exposed to 200 μM H_2_O_2_ for 4 h	24 h treatment: nucleotide mixture 100 μM (AMP:CMP:GMP:UMP = 22.8:25.8:30.2:20.4); individual AMP/CMP/GMP/UMP 50–200 μM; NMN 0.5 mM comparator	ROS, MDA, SOD, senescence markers, mitochondrial, inflammatory endpoints	ROS/MDA ↓; cell viability ↑; senescence markers attenuated. In vitro; NMN is a distinct NAD^+^ precursor.	[76]
Cellular: PC-12 exposed to 200 μM H_2_O_2_ for 4 h	24 h treatment: nucleotide mixture 100 μM; individual AMP/CMP/GMP/UMP 50–200 μM; NMN 0.5 mM positive comparator	ROS, MDA, SOD, GPx, ATP, mitochondrial membrane potential, apoptosis/DNA damage	ROS/MDA ↓; SOD/GPx, ATP, and mitochondrial membrane potential ↑; apoptosis/DNA damage ↓. In vitro neuronal stress model; NMN is pharmacologically distinct.	[59]
Preclinical: male SAMP8/SAMR1 mice; approximately 15 animals/group; 9-month feeding	Standard diet or nucleotides 0.3, 0.6, or 1.2 g/kg; NT-free diet and SAMR1 controls; NMN 0.3 g/kg positive control	BAT MDA, SOD, GPx, UCP1, PGC-1α, PRDM16, AMPK/SIRT1	Low/intermediate doses: antioxidant and thermogenesis-related indices ↑; MDA ↓; high dose not uniformly superior. Recurrent SAMP8 evidence cluster.	[58]
Preclinical: SAMP8/SAMR1 mice; 9-month feeding	Low-, middle-, and high-dose dietary NT groups compared with aging and normal control groups	Skin SOD, MDA, ATP, NAD^+^/NADH, autophagy, aging markers	Changes in redox, mitochondrial, and autophagy-related changes. Tissue aging model; indirect relevance to human insulin resistance.	[77]
Preclinical: 45 SAMP8 mice (15/group) plus 15 SAMR1 mice; 9 months	Standard diet vs. nucleotides 0.3 or 1.2 g/kg	Cardiac GPx, SOD, MDA, ATP, citrate synthase, SDH, AMPK/TFAM-related bioenergetics	GPx/SOD and bioenergetic indices ↑; MDA ↓. Aging cardiac model; same SAMP8 evidence cluster.	[47]
Preclinical: male SAMP8/SAMR1 mice; 9-month intervention	NT-free, normal control, and low/middle/high-nucleotide diets	Testicular Nrf2, SOD, GPx, MDA, testosterone-related pathways	Nrf2/SOD/GPx-related redox indices ↑; MDA ↓; endocrine markers modulated. Organ-specific aging model; limited relevance to adult human insulin resistance.	[78]
Preclinical: aged SAMP8 mice; 9-month intervention	Dietary nucleotide supplementation vs. aging controls	Hepatic antioxidant capacity, glycolysis, ATP, lipid accumulation, fibrosis-related outcomes	Hepatic antioxidant/metabolic indices ↑; lipid accumulation and fibrosis-related changes ↓. Aging model from the same evidence cluster.	[49]
Preclinical: sterlet sturgeon; triplicate groups, three fish/replicate; 10 weeks	0, 1.5, 2.5, 3.5, or 5.0 g/kg of dietary nucleotides	SOD, CAT, GPx, T-AOC, MDA, hepatic/digestive endpoints	Intermediate doses: SOD/CAT/GPx/T-AOC ↑ and MDA ↓; highest dose: MDA ↑. Aquaculture model; strong species limitation.	[79]
Human RCT: 69 adults aged 60–75 years; 3 groups; 10 weeks	Yeast-derived nucleotide preparation (>40% free nucleotides; 250 mg of yeast extract/day) or 5′-monophosphate blend (NF; 150 mg/day) vs. placebo	IL-6, TNF-α, glutathione/redox markers, BDNF, physical and cognitive outcomes	Changes were reported in selected redox, inflammatory, and functional outcomes. Human RCT; participants not selected for metabolic disease; yeast product is multi-component.	[11]
Human RCT: 121 adults aged 60–70 years; 19 weeks; 1:1 allocation	Defined nucleotide preparation 1.2 g/day vs. placebo	Aging-related outcomes, HOMA-IR, safety	HOMA-IR ↓. Surrogate outcome; participants not selected for insulin resistance, type 2 diabetes, or MASLD.	[80]
Preclinical/maternal: 40 multiparous sows (20/group) and suckling piglets	Defined NT mixture, 1 g/kg diet (20% each AMP, UMP, GMP, CMP and IMP), gestation day 85 to lactation day 21	Sow/piglet T-AOC, GSH-Px, TBARS, milk nucleotides, performance	Sow T-AOC ↑; piglet GSH-Px ↑ and TBARS ↓. Maternal/livestock model limits direct translation.	[82]
Preclinical: male NBW and IUGR piglets; 14 matched pairs; postnatal day 7–28	Milk replacer ± 7.409 g of pure NT mixture/kg powder; per 100 kg: AMP 29.6 g, CMP 14.2 g, GMP 40.8 g, IMP 5.8 g, UMP 650.5 g	Plasma/liver/jejunum T-AOC, T-SOD, GPx, GSH/GSSG, MDA; Nrf2, PGC-1α, NRF1, and related redox genes	T-AOC/T-SOD/GPx/GSH:GSSG ↑; hepatic MDA ↓. Developmental IUGR model; UMP-dominant formulation limits adult metabolic translation.	[81]

Abbreviations: AMP, adenosine 5′-monophosphate; AMPK, AMP-activated protein kinase; ATP, adenosine triphosphate; BAT, brown adipose tissue; BDNF, brain-derived neurotrophic factor; CAT, catalase; CMP, cytidine 5′-monophosphate; GMP, guanosine 5′-monophosphate; GPx, glutathione peroxidase; GSH/GSSG, reduced-to-oxidized glutathione ratio; GSH-Px, glutathione peroxidase; H_2_O_2_, hydrogen peroxide; HOMA-IR, homeostatic model assessment of insulin resistance; HUVEC, human umbilical vein endothelial cell; IL-6, interleukin 6; IMP, inosine 5′-monophosphate; IUGR, intrauterine growth restriction; MDA, malondialdehyde; NAD^+^, oxidized nicotinamide adenine dinucleotide; NADH, reduced nicotinamide adenine dinucleotide; NBW, normal birth weight; NF, nucleotide mixture in the form of 5′-monophosphates; NMN, nicotinamide mononucleotide; NRF1, nuclear respiratory factor 1; Nrf2, nuclear factor erythroid 2-related factor 2; PC-12, rat pheochromocytoma cell line; PGC-1α, peroxisome proliferator-activated receptor gamma coactivator 1-alpha; PRDM16, PR domain-containing protein 16; RCT, randomized controlled trial; ROS, reactive oxygen species; SAMP8, senescence-accelerated mouse prone 8; SAMR1, senescence-accelerated mouse resistant 1; SDH, succinate dehydrogenase; SIRT1, sirtuin 1; SOD, superoxide dismutase; T-AOC, total antioxidant capacity; TBARS, thiobarbituric acid-reactive substances; TFAM, mitochondrial transcription factor A; TNF-α, tumor necrosis factor alpha; T-SOD, total superoxide dismutase; UCP1, uncoupling protein 1; UMP, uridine 5′-monophosphate. Symbols: ↑, increase; ↓, decrease.

## Data Availability

No new experimental dataset was generated. The detailed search methodology is provided in Supplementary Data S1. Bibliographic search exports and record-level screening/audit files used for the structured literature search are retained by the authors and can be provided for editorial verification where permitted by database licensing terms; all scientific evidence discussed in the review is available in the cited publications.

## Supplementary Materials

The following supporting information can be downloaded at https://www.mdpi.com/article/10.3390/ijms27188133/s1.

## Abbreviations

The following abbreviations are used in this manuscript:

5′-MPs	5′-monophosphates
ABCG2	ATP-binding cassette subfamily G member 2
ACC	acetyl-CoA carboxylase
ADP	adenosine diphosphate
AKT	protein kinase B
AMP	adenosine 5′-monophosphate
AMPK	AMP-activated protein kinase
AS160	AKT substrate of 160 kDa
ATGL	adipose triglyceride lipase
ATP	adenosine triphosphate
BAT	brown adipose tissue
BCAA	branched-chain amino acid
BDNF	brain-derived neurotrophic factor
cAMP	cyclic adenosine monophosphate
C2C12	mouse myoblast cell line
Caco-2	human colorectal adenocarcinoma cell line
Ca^2+^/Mg^2+^-ATPase	calcium/magnesium-transporting adenosine triphosphatase
CAT	catalase
CDP	cytidine diphosphate
CFU	colony-forming units
CD73	ecto-5′-nucleotidase
CD103	cluster of differentiation 103
CI	confidence interval
CMP	cytidine 5′-monophosphate
ConA	concanavalin A
CNT	concentrative nucleoside transporter
CNT3	concentrative nucleoside transporter 3
COS-7	African green monkey kidney-derived cell line
CPT1α	carnitine palmitoyltransferase 1 alpha
CS	citrate synthase
dAdo	deoxyadenosine
dNMPs	deoxyribonucleoside monophosphates
dAMP	2′-deoxyadenosine 5′-monophosphate
DNA	deoxyribonucleic acid
DOI	digital object identifier
DSS	dextran sulfate sodium
EID	Scopus electronic identifier
ELOVL5	elongation of very long-chain fatty acids protein 5
ENT	equilibrative nucleoside transporter
ENT1	equilibrative nucleoside transporter 1
ENTPD5	ectonucleoside triphosphate diphosphohydrolase 5
FABP1	fatty acid-binding protein 1
FADS2	fatty acid desaturase 2
FOXO1	forkhead box protein O1
G6Pase	glucose-6-phosphatase
GLUT2	glucose transporter type 2
GLUT4	glucose transporter type 4
GMP	guanosine 5′-monophosphate
GPx	glutathione peroxidase
GRAB	G protein-coupled receptor activation-based
GTP	guanosine triphosphate
HDL-C	high-density lipoprotein cholesterol
GSH-Px	glutathione peroxidase
GSH/GSSG	reduced-to-oxidized glutathione ratio
HSL	hormone-sensitive lipase
H_2_O_2_	hydrogen peroxide
HbA1c	glycated hemoglobin
HepG2	human hepatocellular carcinoma cell line
HFD	high-fat diet
hENT1	human equilibrative nucleoside transporter 1
hiPSC-SIEC	human induced pluripotent stem cell-derived small intestinal epithelial cells
HOMA-IR	homeostatic model assessment of insulin resistance
IEL	intraepithelial lymphocyte
HUVEC	human umbilical vein endothelial cell
ICR	Institute of Cancer Research outbred mouse strain
IgA	immunoglobulin A
IgE	immunoglobulin E
IGF-1	insulin-like growth factor 1
IgG	immunoglobulin G
IgM	immunoglobulin M
IL-10	interleukin 10
IL-1α	interleukin 1 alpha
IL-1β	interleukin 1 beta
IL-2	interleukin 2
IL-6	interleukin 6
IL-15	interleukin 15
IL-7	interleukin 7
IRS	insulin receptor substrate
IMP	inosine 5′-monophosphate
IR	insulin resistance
IRS-1	insulin receptor substrate 1
LC-PUFA	long-chain polyunsaturated fatty acid
ISAPP	International Scientific Association for Probiotics and Prebiotics
IUGR	intrauterine growth restriction
LDL-C	low-density lipoprotein cholesterol
LCAT	lecithin–cholesterol acyltransferase
LDH	lactate dehydrogenase
LXRα	liver X receptor alpha
MAPK	mitogen-activated protein kinase
MASLD	metabolic dysfunction-associated steatotic liver disease
MAVS	mitochondrial antiviral-signaling protein
miRNA	microRNA
MCP-1	monocyte chemoattractant protein 1
MDA	malondialdehyde
Na^+^/K^+^-ATPase	sodium/potassium-transporting adenosine triphosphatase
NAD^+^	oxidized nicotinamide adenine dinucleotide
NADH	reduced nicotinamide adenine dinucleotide
NBW	normal birth weight
NDPs	nucleoside diphosphates
NK	natural killer
NF	nucleotide mixture in the form of 5′-monophosphates
NF-κB	nuclear factor kappa B
NMN	nicotinamide mononucleotide
NMPs	nucleoside monophosphates
NRF1	nuclear respiratory factor 1
Nrf2	nuclear factor erythroid 2-related factor 2
NT	nucleotides
NTPDase	ectonucleoside triphosphate diphosphohydrolase
NTPDase1	ectonucleoside triphosphate diphosphohydrolase 1
NTPDase2	ectonucleoside triphosphate diphosphohydrolase 2
NTPDase3	ectonucleoside triphosphate diphosphohydrolase 3
NTPs	nucleoside triphosphates
P1	P1 purinergic receptor
P2Y	P2Y purinergic receptor family
P2X4	purinergic receptor P2X4
P2X7	purinergic receptor P2X7
P2Y2	purinergic receptor P2Y2
p16INK4A	cyclin-dependent kinase inhibitor 2A
p21	cyclin-dependent kinase inhibitor 1A
PC-12	rat pheochromocytoma cell line
PEPCK	phosphoenolpyruvate carboxykinase
PGC-1α	peroxisome proliferator-activated receptor gamma coactivator 1-alpha
PI3K	phosphoinositide 3-kinase
PI3Kγ	phosphoinositide 3-kinase gamma
PKA	protein kinase A
PMID	PubMed identifier
poly-N	polynucleotides
PPARγ	peroxisome proliferator-activated receptor gamma
PRDM16	PR domain-containing protein 16
PRISMA	Preferred Reporting Items for Systematic Reviews and Meta-Analyses
PRISMA-S	PRISMA literature-search extension
PUFA	polyunsaturated fatty acid
RAS	renin–angiotensin system
RCT	randomized controlled trial
RNA	ribonucleic acid
ROS	reactive oxygen species
SA-β-gal	senescence-associated beta-galactosidase
SAMP8	senescence-accelerated mouse prone 8
SAMR1	senescence-accelerated mouse resistant 1
SDH	succinate dehydrogenase
SIRT1	sirtuin 1
SOD	superoxide dismutase
SREBP1c	sterol regulatory element-binding protein 1c
STING	stimulator of interferon genes
T-AOC	total antioxidant capacity
T-SOD	total superoxide dismutase
TALENTs	Targeting Aging and Longevity with Exogenous Nucleotides
TBARS	thiobarbituric acid-reactive substances
TBK1	TANK-binding kinase 1
TCRγδ	gamma-delta T-cell receptor
TFAM	mitochondrial transcription factor A
TFF3	trefoil factor 3
Th1	type 1 helper T cell
TIMP-1	tissue inhibitor of metalloproteinases 1
TGF-β	transforming growth factor beta
TGF-β1	transforming growth factor beta 1
TLR2	Toll-like receptor 2
TLR4	Toll-like receptor 4
TNF-α	tumor necrosis factor alpha
UCP1	uncoupling protein 1
VLDL-C	very-low-density lipoprotein cholesterol
UMP	uridine 5′-monophosphate
UTP	uridine triphosphate
VNUT	vesicular nucleotide transporter
VO_2_max	maximal oxygen uptake
ZO-1	zonula occludens 1
