# Supplementary material for: Dietary Nucleotides as Potential Modulators of Inflammatory and Metabolic Pathways with Implications for Insulin Resistance
Source: Int J Mol Sci. 2026 Sep 12;27(18):8133. doi: 10.3390/ijms27188133 (PMC13607318; doi:10.3390/ijms27188133)
Supplement: Supplementary file 1 [file ijms-27-08133-s001.zip › ijms-4528423-supplementary.pdf]

Supplementary Data S1

Detailed structured literature-search methodology

Dietary nucleotides as potential modulators of inflammatory and metabolic pathways with implications for insulin resistance

Renata Karaś, Urszula E. Binduga, and Konrad A. Szychowski

Note. Reference numbers in this Supplementary Data correspond to the reference list in the main manuscript. Figure 1, retained in the main manuscript, presents the direct dietary and exogenous nucleotide search flow in a diagram informed by PRISMA 2020 and PRISMA-S. The record counts shown in the figure correspond to the updated search performed on 30 August 2026.

S1.1. Information sources and full search strategy

The structured literature search was conducted in PubMed and Scopus without publication year restrictions. The initial search was performed on 1 June 2026. Following peer review, the direct search strategy for dietary and exogenous nucleotide evidence was refined to improve specificity for nutritionally relevant exposures while maintaining sensitivity for eligible studies. The complete refined search was repeated on 30 August 2026 to identify newly published or newly indexed studies and to update the evidence base. The search and screening process was reported with reference to the Preferred Reporting Items for Systematic Reviews and Meta-Analyses (PRISMA) 2020 statement and PRISMA-S where applicable [13–14]; these reporting frameworks improve transparency of the search process without reclassifying the article as a systematic review. No database-level language or publication-type filter was applied.

The final PubMed direct-search strategy used for the 30 August 2026 rerun was: (("dietary nucleotide*"[Title/Abstract] OR "exogenous nucleotide*"[Title/Abstract] OR "oral nucleotide*"[Title/Abstract] OR "sublingual nucleotide*"[Title/Abstract] OR "nucleotide supplement*"[Title/Abstract] OR "nucleotide mixture*"[Title/Abstract] OR "nucleotide preparation*"[Title/Abstract] OR "nucleotide enriched"[Title/Abstract] OR "nucleotide-enriched"[Title/Abstract] OR "nucleotide supplemented"[Title/Abstract] OR "nucleotide-supplemented"[Title/Abstract] OR "nucleotide enriched diet*"[Title/Abstract] OR "nucleotide-enriched diet*"[Title/Abstract] OR "nucleotide supplemented diet*"[Title/Abstract] OR "nucleotide-supplemented diet*"[Title/Abstract] OR "nucleotide enriched formula*"[Title/Abstract] OR "nucleotide-enriched formula*"[Title/Abstract] OR "nucleotide supplemented formula*"[Title/Abstract] OR "nucleotide-supplemented formula*"[Title/Abstract] OR "nucleotide enriched feed*"[Title/Abstract] OR "nucleotide-enriched feed*"[Title/Abstract] OR "nucleotide supplemented feed*"[Title/Abstract] OR "nucleotide-supplemented feed*"[Title/Abstract] OR "yeast derived nucleotide*"[Title/Abstract] OR "yeast-derived nucleotide*"[Title/Abstract] OR "dietary ribonucleotide*"[Title/Abstract] OR "ribonucleotide supplement*"[Title/Abstract] OR "dietary nucleic acid*"[Title/Abstract] OR "exogenous nucleic acid*"[Title/Abstract] OR "oral nucleic acid*"[Title/Abstract] OR "food-derived nucleic acid*"[Title/Abstract] OR "food derived nucleic acid*"[Title/Abstract] OR "food-derived DNA"[Title/Abstract] OR "food derived DNA"[Title/Abstract] OR "dietary DNA"[Title/Abstract] OR "food-derived RNA"[Title/Abstract] OR

"dietary RNA"[Title/Abstract]) AND ("insulin resistance"[Title/Abstract] OR "insulin sensitivity"[Title/Abstract] OR insulin[Title/Abstract] OR "glucose metabolism"[Title/Abstract] OR "glucose homeostasis"[Title/Abstract] OR "glucose tolerance"[Title/Abstract] OR glucose[Title/Abstract] OR glycem*[Title/Abstract] OR glycaem*[Title/Abstract] OR "lipid metabolism"[Title/Abstract] OR lipogenesis[Title/Abstract] OR lipogenic[Title/Abstract] OR "fatty acid metabolism"[Title/Abstract] OR lipid*[Title/Abstract] OR liver[Title/Abstract] OR hepatic[Title/Abstract] OR steatosis[Title/Abstract] OR MASLD[Title/Abstract] OR adipos*[Title/Abstract] OR obes*[Title/Abstract] OR inflamm*[Title/Abstract] OR immun*[Title/Abstract] OR "oxidative stress"[Title/Abstract] OR antioxidant*[Title/Abstract] OR redox[Title/Abstract] OR microbiot*[Title/Abstract] OR microbiom*[Title/Abstract] OR intestin*[Title/Abstract] OR gut[Title/Abstract] OR "intestinal barrier"[Title/Abstract] OR "gut barrier"[Title/Abstract] OR colitis[Title/Abstract] OR mucos*[Title/Abstract] OR ulcer*[Title/Abstract] OR "intestinal injury"[Title/Abstract] OR "intestinal repair"[Title/Abstract] OR absorp*[Title/Abstract] OR uptake[Title/Abstract] OR hydrolys*[Title/Abstract] OR bioavailab*[Title/Abstract] OR salvage[Title/Abstract] OR growth[Title/Abstract] OR diarrh*[Title/Abstract] OR safety[Title/Abstract])). The equivalent final Scopus direct-search strategy was: TITLE-ABS-KEY(("dietary nucleotide*" OR "exogenous nucleotide*" OR "oral nucleotide*" OR "sublingual nucleotide*" OR "nucleotide supplement*" OR "nucleotide mixture*" OR "nucleotide preparation*" OR "nucleotide-enriched" OR "nucleotide-supplemented" OR "yeast-derived nucleotide*" OR "yeast derived nucleotide*" OR "dietary ribonucleotide*" OR "ribonucleotide supplement*" OR "dietary nucleic acid*" OR "exogenous nucleic acid*" OR "oral nucleic acid*" OR "food-derived nucleic acid*" OR "food derived nucleic acid*" OR "food-derived DNA" OR "food derived DNA" OR "dietary DNA" OR "food-derived RNA" OR "dietary RNA" OR (nucleotide* W/3 diet*) OR (nucleotide* W/3 oral) OR (nucleotide* W/3 supplement*) OR (nucleotide* W/3 formula*) OR (nucleotide* W/3 feed*) OR (nucleotide* W/3 nutrition*) OR (nucleotide* W/3 sublingual) OR (ribonucleotide* W/3 diet*) OR (ribonucleotide* W/3 oral) OR (ribonucleotide* W/3 supplement*) OR (ribonucleotide* W/3 formula*) OR (ribonucleotide* W/3 feed*) OR ("nucleic acid*" W/3 diet*) OR ("nucleic acid*" W/3 oral) OR ("nucleic acid*" W/3 exogenous) OR ("nucleic acid*" W/3 food*)) AND ("insulin resistance" OR "insulin sensitivity" OR insulin OR "glucose metabolism" OR "glucose homeostasis" OR "glucose tolerance" OR glucose OR glycem* OR glycaem* OR "lipid metabolism" OR lipogenesis OR lipogenic OR "fatty acid metabolism" OR lipid* OR liver OR hepatic OR steatosis OR MASLD OR adipos* OR obes* OR inflamm* OR immun* OR "oxidative stress" OR antioxidant* OR redox OR microbiot* OR microbiom* OR intestin* OR gut OR "intestinal barrier" OR "gut barrier" OR colitis OR mucos* OR ulcer* OR "intestinal injury" OR "intestinal repair" OR absorp* OR uptake OR hydrolys* OR bioavailab* OR salvage OR growth OR diarrh* OR safety)). The separate contextual purinergic-signalling search was kept outside the direct nutritional-intervention flow and used: PubMed, ("extracellular ATP"[Title/Abstract] OR "extracellular adenosine"[Title/Abstract] OR purinergic[Title/Abstract] OR P2X7[Title/Abstract] OR P2Y2[Title/Abstract] OR ENTPD5[Title/Abstract] OR NTPDase3[Title/Abstract] OR ENT1[Title/Abstract]) AND ("glucose metabolism"[Title/Abstract] OR "lipid metabolism"[Title/Abstract] OR adipogenesis[Title/Abstract] OR thermogenesis[Title/Abstract] OR obesity[Title/Abstract] OR MASLD[Title/Abstract] OR "insulin resistance"[Title/Abstract]); Scopus, TITLE-ABS-KEY(("extracellular ATP" OR "extracellular adenosine" OR purinergic OR P2X7 OR P2Y2 OR ENTPD5 OR NTPDase3 OR ENT1) AND ("glucose metabolism" OR "lipid metabolism" OR adipogenesis OR thermogenesis OR obesity OR MASLD OR "insulin resistance")).

To reduce the risk of missing eligible evidence during revision, backward citation chasing was performed from retained primary studies and key reviews. Targeted forward and recent literature checks were also undertaken for direct dietary, oral, sublingual, maternal, intestinal absorption, and food-derived nucleic acid interventions, including terminology variants not consistently captured by the principal query. A separate contextual purinergic signalling search and methodological or background searches were used where needed for PRISMA reporting, general metabolic context, food composition, transport biology, and interpretation. These supplementary sources were assessed individually for relevance and citation-to-claim accuracy and were not added to the PubMed and Scopus direct search record counts. The final bibliography therefore should not be interpreted as a numerical continuation of the direct search flow. The 2026 Web of Science Core Collection bibliometric map by Tian and Xu was also examined as an external completeness check. It covered a broader exogenous nucleotide literature from 2000 through 2025 and analysed 710 records [15]. This count was not incorporated into the present search flow because the database, search fields, eligibility scope, and bibliometric objective differed. Full texts were checked when formulation, dose, duration, sample size, sex, experimental model, route, comparator, or exposure classification could not be established reliably from the title and abstract.

S1.2. Detailed eligibility criteria

Eligible primary evidence comprised peer-reviewed original human, animal, cellular, analytical, and other relevant experimental or observational studies that evaluated a nutritionally or experimentally relevant exogenous exposure to nucleotides. Eligible interventions included purified 5′-monophosphates, defined nucleotide mixtures, yeast-derived nucleotide preparations, nucleotide-supplemented formulas or feeds, oral or sublingual nucleotide preparations, and clearly identified dietary or food-derived nucleic acid exposures. Nucleosides or nucleobases were retained as a separate adjacent exposure category when they addressed absorption, salvage, metabolism, or a mechanistic question that was directly relevant to the interpretation of dietary nucleotides. Eligible outcomes included intestinal hydrolysis, absorption, bioavailability, insulin and glucose regulation, lipid and hepatic metabolism, inflammatory or immune responses, redox homeostasis, intestinal barrier structure or function, mucosal injury or repair, colitis related outcomes, microbiota, growth, diarrhoea, and safety. No study was excluded solely because of publication year. Conference abstracts or proceedings without a full peer reviewed report, editorials, commentaries, protocols, non-peer-reviewed reports, reviews, systematic reviews, and meta-analyses were excluded from the primary evidence set. Reviews and meta analyses could still be used for terminology, background, and backward citation chasing. Pharmacological nucleoside or nucleotide analogues, oligonucleotide drugs, parenteral interventions without nutritional relevance, and records in which nucleotide or nucleic acid terminology referred only to sequence variation, single-nucleotide polymorphisms, nucleotide-binding proteins, nucleotide synthesis, gene delivery tools, or another non-nutritional molecular or genetic context were excluded from direct supplementation evidence. Endogenous extracellular purinergic signalling studies were searched separately and were used only as contextual mechanistic evidence.

S1.3. Study selection, data extraction, and citation verification

For each primary study discussed in detail, study characteristics were verified from the full text whenever reported. These included species or cell model, strain, sex, age or developmental stage, sample size and experimental unit, nucleotide source and composition, dose or concentration, route, comparator, intervention duration, challenge model where applicable, principal outcomes, and major translational limitations. Neutral, null, and adverse findings were retained to reduce selective emphasis on positive results. The 518 records retained after staged title and abstract screening formed the pool for further eligibility assessment and should not be interpreted as the number of studies included in the narrative synthesis. Eligibility was assessed on the basis of exposure, model, outcome, and publication type. Full texts were examined for records considered potentially eligible and whenever eligibility or important design characteristics could not be determined reliably from the title and abstract. Together with the supplementary source identification routes described in Section S1.1, this process defined the evidence base used for the narrative synthesis. The final bibliography contains 161 cited publications, of which 152 are original research reports and 9 are secondary, methodological, bibliometric, review, reporting guideline, or consensus and background publications. This publication-level classification is separate from the direct search record flow described in Section S1.4. The 152 original reports do not represent 152 independent studies because related publications from the same cohort or experimental programme were retained only when they contributed additional endpoints. All 161 cited publications underwent citation-to-claim verification.

S1.4. Screening, deduplication, and record-flow details

The updated direct dietary and exogenous nucleotide search identified 2,266 database records, with 541 from PubMed and 1,725 from Scopus. Automated deduplication used digital object identifiers, PubMed identifiers, and Scopus electronic identifiers where available, followed by exact concordance of the normalised title and publication year. Removal of 528 duplicates left 1,738 unique records for software-assisted title and abstract screening. Initial scope and publication-type screening excluded 418 non-primary publications, 206 pharmacological or oligonucleotide drug records, and 1 other low-relevance record, leaving 1,113 records. Exposure-specific title and abstract screening then excluded 595 records in which a clearly relevant dietary or exogenous nucleotide exposure was not identified. The remaining 518 records were retained for further eligibility assessment, with targeted full-text verification performed when required. These values describe the direct search record flow and do not represent the number of studies included in the narrative synthesis. Additional sources identified through the supplementary routes described in Section S1.1 were not included in these counts. The separate purinergic search also remained outside this flow because it addressed endogenous extracellular signalling rather than the direct nutritional intervention question. Because this article remains a narrative review, Figure 1 presents the direct search and screening flow in a diagram informed by PRISMA 2020 and PRISMA-S rather than as a formal systematic review inclusion diagram.

S1.5. Evidence classification and synthesis

Potentially overlapping publications were evaluated at the level of the underlying cohort or experimental programme rather than treated automatically as independent confirmation. The Fu et al. multi-omics papers are secondary analyses of the TALENTs cohort. The older thioacetamide and cirrhosis

studies from the Granada group form a related experimental cluster, the Cai et al. alcohol liver papers represent closely related experiments, and several later SAMP8 studies originate from the same broader ageing nucleotide research network. These reports were retained when they contributed additional endpoints but were not interpreted as independent replication.

To avoid combining biologically and pharmacokinetically distinct exposures, evidence was classified into five categories. These comprised purified oral 5′-monophosphate nucleotides, defined nucleotide mixtures and nucleotide-supplemented formulas, feeds, or yeast-derived preparations, nucleosides and nucleobases, dietary nucleic acids or purine-rich foods, and endogenous extracellular purinergic signalling. The fifth category was used only as contextual mechanistic evidence and was not interpreted as evidence that oral nucleotide supplementation modifies extracellular ATP, ADP, AMP, or adenosine signalling in human metabolic tissues. Multi-component products, food-derived RNA or DNA, and studies using nicotinamide mononucleotide were identified explicitly because their effects cannot be attributed unambiguously to conventional dietary 5′-monophosphates.
